# Field Evaluation of Diagnostic Test Sensitivity and Specificity for Salmonid Alphavirus (SAV) Infection and Pancreas Disease (PD) in Farmed Atlantic salmon (*Salmo salar L*.) in Norway Using Bayesian Latent Class Analysis

**DOI:** 10.3389/fvets.2019.00419

**Published:** 2019-11-28

**Authors:** Mona Dverdal Jansen, Mario Guarracino, Marianne Carson, Ingebjørg Modahl, Torunn Taksdal, Hilde Sindre, Edgar Brun, Saraya Tavornpanich

**Affiliations:** ^1^Norwegian Veterinary Institute, Oslo, Norway; ^2^Royal Veterinary College, London, United Kingdom

**Keywords:** salmonid alphavirus, pancreas disease, Atlantic salmon, Bayesian latent class analysis, real-time RT-PCR, diagnostic sensitivity, diagnostic specificity

## Abstract

Salmonid alphavirus (SAV) is the OIE-listed, viral cause of pancreas disease (PD) in farmed Atlantic salmon. SAV is routinely detected by PCR–methods while typical histopathological lesions are additionally used to confirm the diagnosis. Field evaluation of diagnostic test performance is essential to ensure confidence in a test's ability to predict the infection or disease status of a target animal. For most tests used in aquaculture, characteristics like sensitivity (Se) and specificity (Sp) at the analytical level may be known. Few tests are, however, evaluated at the diagnostic level according to the OIE standard. In the present work, we estimated diagnostic test sensitivity (DSe) and diagnostic test specificity (DSp) for five laboratory tests used for SAV detection. As there is no gold standard, the study was designed using Bayesian latent class analysis. Real-time RT-PCR, cell culture, histopathology, virus neutralization test, and immunohistochemistry were compared using samples taken from three different farmed Atlantic salmon populations with different infection status; one population regarded negative, one in an early stage of infection, and one in a later stage of infection. The average fish weight in the three populations was 2.0, 1.6, and 1.5 kg, respectively. The DSe and DSp of real-time RT-PCR is of particular interest due to its common use as a screening tool. The method showed high DSe (≥0.977) and moderate DSp (0.831) in all 3-populations models. The results further suggest that a follow-up test of serum samples in real-time RT-PCR negative populations may be prudent in cases where epidemiological information suggest a high risk of infection and where a false negative result is of high consequence. This study underlines the need to choose a test appropriate for the purpose of the testing. In the case of a weak positive PCR-result, a follow-up test should be conducted to verify the presence of SAV. Cell culture showed high DSe and DSp and may be used to verify viral presence.

## Introduction

The ability to reliably detect the presence of important infectious disease agents in aquatic animal populations is essential for such diverse reasons as disease surveillance and international trade. All member countries of the World Organization for Animal Health (OIE) are obliged to provide timely and transparent information on its situation on listed animal diseases, information that plays an important part in relation to international trade and trade partner trust. The evaluation of a diagnostic test for its performance is essential to ensure confidence in the test's ability to accurately predict the infection or exposure status of the target animal (1). An important stage in OIE's test development and validation pathway is the validation of a test's diagnostic sensitivity (DSe) and diagnostic specificity (DSp) under field conditions (2), using a reference or gold standard test i.e., a diagnostic test which correctly identifies all true positive animals as positive and all true negative animals as negative.

The DSe in a veterinary context is the conditional probability that an infected animal will be test-positive, while DSp is the conditional probability that an uninfected animal will be test-negative (1). A gold standard test does not always exist or might not be available for field conditions. One alternative analytical tool for estimation of DSe and DSp is Bayesian latent class analysis (3, 4). The Bayesian approach for estimating diagnostic test accuracy does not require knowledge of true infection status and allows for the incorporation of prior scientific knowledge, such as disease prevalence in study populations, when specifying model parameters. Both prior information and new, collected data can then be used to assess diagnostic test performance. The models provide posterior distributions of the parameters of interest e.g., the median and 95% probability interval for test DSe and DSp.

The test performance (DSe and DSp), which are the properties of the diagnostic tool, are not subject to change with prevalence. However, DSe and DSp can be influenced by individual characteristics, and so the estimates can differ when applied to populations under different stages of infection e.g., infection during a long latent period or chronic infection. Bayesian latent class analysis is a robust method which has been widely used for the estimation of diagnostic test performance when a gold standard test does not exist. It is also the only method for combining existing knowledge and expert opinions into equations. However, there have been very few uses of the method in the field of aquatic animal diseases (5–8).

The Norwegian salmonid aquaculture industry is largely relying on foreign markets for its products and according to the Norwegian Seafood Council, one million metric tons of salmon was exported from Norway in 2017 (9). One of the major disease challenges affecting the Norwegian production is the OIE-listed viral agent salmonid alphavirus (SAV), with around 140–160 new grow-out populations being infected every year (10). There are six known genotypes of SAV (11), and the geographical distribution of the two endemic SAV-genotypes present in Norway, SAV3 and marine SAV2, are partially overlapping (12–14). As reviewed by Jansen et al. (14) infected fish commonly present with reduced appetite and abnormal swimming behavior prior to the onset of mortality. Post-mortem inspection commonly reveals empty intestines, fecal casts and signs of circulatory disturbance. On histological examination, major lesions include loss of exocrine pancreatic tissue, cardiac myocytic necrosis, and inflammation and degeneration of skeletal muscle. While mortality levels have been reported to be significantly higher in populations affected by SAV3 than marine SAV2 both during experimental- and field studies (15, 16), the histopathological changes appear similar regardless of underlying SAV genotype (16, 17). A proportion of surviving fish may develop severe fibrosis of the peri-acinar tissue and subsequently fail to grow (17). Clinical signs and macroscopic changes are not in themselves pathognomonic, and thus a diagnosis depends on the application of laboratory diagnostic tests (17). Subclinical infections of SAV occur (18, 19), and variation in prevalence due to SAV genotype has been reported. In Norway, infections with marine SAV2 are reported to generate a higher proportion of subclinical cases than SAV3 infections (15).

The OIE disease card for SAV lists a number of laboratory tests that have been deemed appropriate for diagnostic purposes for detection of SAV or histopathological changes consistent with SAV infection (20). These include real-time RT-PCR, conventional RT-PCR (with sequencing for genotyping), immunohistochemistry, histopathology, serum neutralization assay, and isolation of virus in cell culture. A serum-based selective precipitation reaction (SPR) has also been recently described (21), however this is currently not in use in Norway and is not listed on the OIE disease card.

There has been a lack of scientific field-validation of the diagnostic tests used in conjunction with SAV detection and histopathological changes consistent with SAV infection in Norway. In the autumn of 2017 Norway introduced a new screening protocol where all salmonid seawater grow-out sites that are stocking fish are sampled monthly for the detection of SAV by real-time RT-PCR until SAV is eventually detected or the population is slaughtered. As a result, an evaluation of the DSe and DSp of the applied diagnostic tests in general, and the real-time RT-PCR in particular, is of interest to a range of stakeholders including the government, the competent authority, and the industry. The aim of this study was therefore to evaluate the field performance of the available diagnostic tests for SAV in Norway assuming there was no gold standard.

## Materials and Methods

### Source Populations and Sampling Procedures

Three Atlantic salmon seawater grow-out sites were included in this study. The sites were purposely selected on the basis of the assumed infection status of their populations; one non-infected population and two SAV-infected populations at different stages of infection.

Site 1 was located in the eastern part of the northernmost county of Norway (Finnmark), a county with no SAV detections since 2013 and without any previous SAV detections in its eastern part. This site was therefore assumed to stock a non-infected population, and the site was sampled in May 2017. At the time of sampling, the site stocked ~1.5 million fish at an average weight of ~2 kg.

Site 2 was located in south-western Norway (Hordaland county), an area that has a high prevalence of SAV3-infected sites. Following routine disease investigation procedures, the site was diagnosed with SAV3 PD (SAV3 detection and histopathological changes indicative of SAV infection in the same individual) in May 2017 and was sampled for this study in the same month. The fish population had been vaccinated with a commercially available SAV vaccine (based on SAV1). The fish were transferred to sea in September 2016, and at time of sampling the site stocked ~1 million fish at an average weight of ~1.6 kg. There was no major clinical disease outbreak at the time of sampling.

Site 3 was located in mid-Norway (Møre and Romsdal county), an area that has a high prevalence of SAV2-infected sites. The site was diagnosed with SAV2 PD (SAV2 detection and histopathological changes indicative of SAV infection in the same individual) in February 2017, and sample collection was performed in June 2017. The fish population was not vaccinated against SAV. The fish were transferred to sea in September 2016, and at time of sampling, the site stocked ~630,000 fish at an average weight of ~1.5 kg. There was no major clinical disease outbreak after the diagnosis date until the time of sampling.

Sampling procedures were similar at all three sites. One cage was selected at each site; a randomly selected cage at Site 1 and a cage where PD had been diagnosed at Site 2 and Site 3. One hundred fish were convenience sampled from each cage using a seine net. All fish were sedated and subsequently humanely euthanized by a blow to the head. Based on the sampling procedures outlined by the Norwegian Veterinary Institute (22), the following set of samples were aseptically collected from each fish: tissue samples in RNAlater^TM^ (head kidney, heart ventricle), in 10% buffered formalin (NVI, product number 30.054.08) (gill, head kidney, heart ventricle, liver, pancreas, red and white muscle, spleen) and in viral transport medium (NVI, product number 50.008.00) (head kidney, heart ventricle), and blood samples on standard tubes for serological investigation (BD Plastipak^TM^ 2 ml syringes with 21G needles). Samples were stored on ice until arrival at the laboratory storage facilities.

### Diagnostic Tests

Samples from each fish were tested at the Norwegian Veterinary Institute laboratory for the presence of SAV, SAV antibodies or for typical histopathological lesions consistent with SAV infection by five different diagnostic tests, namely real-time RT-PCR, isolation in cell culture, virus neutralization test, histopathology, and immunohistochemistry. See Table 1 for details on the test purpose, target tissue, and classification criteria of test-positive and test-negative samples.

**Table 1:** Test purpose, target tissue and criteria for positive and negative results for the five evaluated diagnostic tests.

**Test**	**Test purpose**	**Target tissue**	**Positive result**	**Negative result**
PCR	Detection of SAV-RNA	Heart ventricle Head kidney	Ct-value < 40	Ct-value ≥ 40 No Ct-value obtained
CELL	Isolation of SAV	Heart ventricle Head kidney	SAV infection of cells	No SAV infection of cells
NT	Detection of antibodies/neutralizing activity against SAV	Serum	Virus neutralization at 1:20 dilution only or at both 1:20 and 1:80 dilutions	No virus neutralization
HIST	Detection of pathological lesions consistent with SAV infection	Heart ventricle Pancreas Red and white muscle	Lesions consistent with, or indicative of, SAV infection	No lesions indicative of SAV infection
IHC	Detection of SAV	Pancreas	Positive staining of necrotic exocrine pancreatic cells	No staining of exocrine pancreatic cells

#### Real-Time RT-PCR (PCR)

For the detection of SAV-RNA in heart and kidney tissues, a real-time RT-PCR assay was used to test for the presence of the conserved SAV Qnsp1 gene as described by Hodneland and Endresen (23), with some modifications. Briefly, nucleic acids were extracted using the NucliSens^®^ easyMAG™ (bioMérieux) system according to the manufacturer's instructions. The Brilliant III Ultra-Fast QRT-PCR (Agilent Technologies) master mix was used according to the manufacturer's instructions and amplification was performed using a Stratagene Mx3005P system (Agilent Technologies) over 40 cycles. Reactions with a cycle threshold (Ct) < 40 were considered positive. The Qnsp1 assay is capable of detecting all currently known SAV genotypes and, as a result, all SAV-positive populations will have the SAV genotype determined by subsequent sequencing.

#### Isolation in Cell Culture (CELL)

SAV isolation from tissue samples was performed as previously described by Jansen et al. (24) and inoculated in 1:10 and 1:80 dilutions onto Chinook salmon embryo culture (CHSE-214) plates which had been grown at 20°C. After 2 weeks of incubation at 15°C, the plates were freeze-thawed and the cell lysate were inoculated on new cell cultures and incubated for a further 2 weeks. As the Norwegian field isolates of SAV2 and SAV3 rarely induce CPE in CHSE-214 cells, indirect immunofluorescence antibody test (IFAT) was used to visualize SAV-infected cells. Briefly, a 96-well CHSE plate was inoculated with cell culture supernatants and incubated at 15°C for 10 days. After fixation in 80% acetone, 50 μl of diluted SAV-specific mouse monoclonal antibody 17H23 directed against the E2 glycoprotein (25) was added per well and incubated for 1 h, followed by subsequent incubation for 1 h with diluted secondary biotinylated goat anti-mouse IgG antibody (DAKO) before the final incubation with streptavidin-fluorescein isothiocyanate (FITC) conjugate (eBioscience). Stained cell cultures were examined on an inverted fluorescence microscope. Positive samples were those with two or more fluorescent cells in at least two parallel wells and where the negative controls did not show any fluorescent cells.

#### Virus Neutralization Test (NT)

The presence of SAV neutralizing antibodies in fish serum samples was tested for using a virus neutralization test as described by Graham et al. (26) with modifications as described by Taksdal et al. (27). Briefly, plasma samples diluted to 1:20 and 1:80 were mixed with equal parts of a SAV1 reference strain (F93-125, Ireland) and incubated for 2 h at room temperature before inoculation onto 96-well CHSE plates. A 1:20 serum dilution without virus was used as the control. Plates were incubated for 12 days before IFAT staining, as described in section Isolation in Cell Culture (CELL). Virus neutralization at antibody titres ≥1:20 were considered positive.

#### Histopathological Examination (HIST)

Tissue samples were examined for histopathological changes consistent with SAV infection as described by Taksdal et al. (16). Samples were fixed with formaldehyde, and 4–6 μm hemotoxylin and eosin stained sections were examined under a light microscope. Samples were classified as either showing (1) changes consistent with SAV infection, (2) changes that might indicate SAV infection, or (3) no changes consistent with SAV infection. Samples with changes consistent with SAV infection or changes that might indicate SAV infection were considered positive.

#### Immunohistochemistry (IHC)

Detection of SAV in tissues by immunohistochemistry was performed as described by Taksdal et al. (27). Briefly, after blocking non-specific antibody binding sites, primary anti-SAV mAb was diluted to 1:3,000 and added to the tissue samples before overnight incubation at room temperature. Tissues were incubated with a 1:300 dilution of secondary biotinylated rabbit anti-mouse IgG and IgM before further incubation with a 1:500 dilution of streptavidin-alkaline phosphatase complex at room temperature. Tissue sections were stained with fast red salt in fast red substrate solution and examined under a light microscope for antibody-antigen binding. Samples with positive staining of necrotic exocrine pancreatic cells were considered positive.

### Estimation of Test Diagnostic Sensitivity and Specificity

#### Bayesian Latent Class Analysis

Bayesian latent class analysis was used to estimate the test performance characteristics (DSe and DSp) of the five tests, assuming that none of these tests could be considered as a gold standard. The model was modified from the study of bacterial kidney disease test accuracy (6) and the code was provided by Jaramillo et al. (6). The study followed the guidelines for diagnostic test evaluation as described by Laurin et al. (28) and consulted the standards for the reporting of diagnostic accuracy studies that use Bayesian latent class models as described by Kostoulas et al. (29).

In this study, non-informative priors were assumed for the sensitivity and specificity of all five tests considering that the test characteristics have not previously been established and was implemented using a flat beta distribution: beta (1,1). The existing knowledge of SAV infection status was incorporated as informative priors for Site 1 and Site 2 prevalence. The fish health veterinarians responsible for health monitoring on the study sites were asked to provide the most likely value, and lower or upper limit of the site prevalence, before the samples were collected. The prior for Site 1 SAV-prevalence reflects the assumption that the site was likely to be uninfected, and the most likely number of positive fish would be 1 in 100,000 fish, with an upper limit of 10 in 100,000 fish. The prior of site 2 prevalence was chosen to reflect the knowledge that the site was infected. Site 2 was sampled in the same month as the diagnosis and could therefore either be heavily infected, if infection had been undetected for a while, or have an increasing prevalence. The most likely prevalence was assumed to be 60% with a large uncertainty ranging between 5 and 95%. Site 3 was sampled 4 months after diagnosis and could therefore either be moderately infected or have a low infection prevalence due to recovery. Due to this uncertainty about the most likely prevalence value for Site 3, a non-informative prior was assumed. The priors of Site 1, Site 2, and Site 3 prevalence were modeled using a beta distribution of beta (2, 100,000), beta (6,4), and beta (1,1), respectively (Table 2).

**Table 2:** Prior information used in the models for site prevalence and diagnostic sensitivity and specificity of all five tests.

**Parameter**	**Expert opinion**			**Prior distribution**
	**Most likely**	**Lower limit**	**Upper limit**	
Site 1 prevalence	1/100,000	–	10/100,000	Beta(2,100,000)
Site 2 prevalence	60%	5%	95%	Beta(6,4)
Site 3 prevalence	–	–	–	Beta(1,1)
DSe of all tests	–	–	–	Beta(1,1)
DSp of all tests	–	–	–	Beta(1,1)

In this study, seven models with different number of tests and number of populations were evaluated. The main model (model 1) was a model with 3-populations and 5-tests where the samples from all sites and all tests were included. Models 2 and 3 were models with 3-populations and 4-tests, while model 4 was a model with 3-populations and 3-tests. Models 5, 6, 7 were models with 2-populations and 5-tests. Details of which populations and tests that were included in each model are presented in Table 3. For biological reasons, potential conditional dependencies between PCR and CELL, and between HIST and IHC, were investigated by estimation of covariance of DSe and DSp for each pair of the tests when possible.

**Table 3:** List of the seven evaluated models with their prior distributions, and the changes to the model priors for sensitivity analysis.

**Model**	**Populations**	**Tests**	**Defaults priors**				
			**Site 1 prevalence**	**Site 2 prevalence**	**Site 3 prevalence**	**DSe of all tests**	**DSp of all tests**
1	Sites 1,2,3	PCR, CELL, NT, HIST, IHC	Beta(2,100,000)	Beta(6,4)	Beta(1,1)	Beta(1,1)	Beta(1,1)
2	Sites 1,2,3	PCR, CELL, HIST, IHC	Beta(2,100,000)	Beta(6,4)	Beta(1,1)	Beta(1,1)	Beta(1,1)
3	Sites 1,2,3	PCR, CELL, NT, HIST	Beta(2,100,000)	Beta(6,4)	Beta(1,1)	Beta(1,1)	Beta(1,1)
4	Sites 1,2,3	PCR, CELL, HIST	Beta(2,100,000)	Beta(6,4)	Beta(1,1)	Beta(1,1)	Beta(1,1)
5	Sites 2, 3	PCR, CELL, NT, HIST, IHC	n/a	Beta(6,4)	Beta(1,1)	Beta(1,1)	Beta(1,1)
6	Sites 1, 2	PCR, CELL, NT, HIST, IHC	Beta(2,100,000)	Beta(6,4)	n/a	Beta(1,1)	Beta(1,1)
7	Sites 1, 3	PCR, CELL, NT, HIST, IHC	Beta(2,100,000)	n/a	Beta(1,1)	Beta(1,1)	Beta(1,1)
**Model**		**Sensitivity analysis (SA)**	**Changes in priors**				
1		SA1	–	Beta(1,1)	–	–	–
1		SA2	Beta(6,100,000)	Beta(1,1)	–	–	–
2		SA3	–	Beta(1,1)	–	–	–
2		SA4	Beta(6,100,000)	Beta(1,1)	–	–	–
3		SA5	–	Beta(1,1)	–	–	–
3		SA6	Beta(6,100,000)	Beta(1,1)	–	–	–
4		SA7	–	Beta(1,1)	–	–	–
4		SA8	Beta(6,100,000)	Beta(1,1)	–	–	–

Model convergence was assessed using graphical diagnostics by running the models with multiple chains of different initial values and checking the trace plots for each parameter. Convergence was further tested for all parameters by Gelman-Rubin statistics (R-hat) < 1.1 (30) using the CODA package in R (https://cran.r-project.org/web/packages/coda/coda.pdf) and Bayesian *p*-value was estimated to assess the model goodness-of-fit. The models were coded in OpenBUGS, v3.2.3 rev 1012 (31). Each model was run for 100,000 iterations of which the first 10,000 iterations were discarded as burn-in. Thinning by 10 was applied to eliminate autocorrelation. Model outputs are presented as the posterior median and 95% probability intervals (2.5th and 97.5th percentiles) and as boxplots of the posterior estimates of site prevalence, DSe and DSp.

#### Difference of DSe and DSp Between Tests

Differences beyond chance of the DSe and DSp between tests were assessed using a built-in step function in OpenBUGS to estimate the probability of the differences between tests for which a probability >0.05 was considered significant. The process was performed using default values of model 1, and focused on selected pairs of tests for which the difference was not determined by visualization alone.

#### Sensitivity Analysis

The effect of changing priors on the estimated DSe and DSp of all tests was evaluated by replacing prior of Site 2 with non-informative prior, and shifting the prior distribution of Site 1 prevalence by 30% from the median default value (Table 3).

### Ethical Approval

This study was performed in accordance with the ethical guidelines of the Norwegian Veterinary Institute. All fish were sedated and humanely euthanized prior to sample collection in accordance with standard procedures.

## Results

### Summary of Test Results

In total, 268 of the 300 collected samples were analyzed and included in the study, while the remaining 32 samples were excluded due to a lack of relevant tissue type or inadequate quality of preservation.

An overview of the sample results for each site, and all sites combined, is presented in Table 4. Test results varied across the three sites and between the five tests. For Site 1, no samples were test-positive by any of the tests. For Site 2, 89% of samples were positive by PCR, followed by CELL (81%), histopathology (56%), NT (6%) and IHC (4%). For Site 3, 53% of samples tested positive by NT, followed by PCR (30%), HIST (5%), and CELL (1%). No samples from Site 3 tested positive by IHC.

**Table 4:** Summary of samples tested using PCR, CELL, NT, HIST and IHC in three Norwegian fish farms, Sites 1–3, investigated for PD in *Salmo salar* L.

	**Number of test positive samples (%)**					
	**Included**	**PCR**	**CELL**	**NT**	**HIST**	**IHC**
	**samples**	**Ct < 40 (%)**	**+ (%)**	**+ (%)**	**+ (%)**	**+ (%)**
All sites	268	101 (38)	67 (25)	56 (21)	50 (19)	3 (1)
Site 1	91	0 (0)	0 (0)	0 (0)	0 (0)	0 (0)
Site 2	81	72 (89)	66 (81)	5 (6)	45 (56)	3 (4)
Site 3	96	29 (30)	1 (1)	51 (53)	5 (5)	0 (0)

The combination of all obtained test results in the study is shown in Table 5. For samples from Site 2, which was assumed to have the highest prevalence of infected individuals in the study, 90% of samples were classified as positive by at least one test. In comparison, 55% of samples from Site 3 were found to test positive by at least one test.

**Table 5:** Diagnostic test result combinations and associated results for PCR, CELL, NT, HIST, and IHC across three Norwegian fish farms, Sites 1–3, investigated for PD in *Salmo salar* L.

		**Test combinations**												**Total**
Test	PCR	+	+	+	+	+	+	+	+	–	–	–	–	
	CELL	+	+	+	+	+	–	–	–	–	–	–	–	
	NT	+	+	–	–	–	+	+	–	+	+	–	–	
	HIST	+	–	+	+	–	+	–	–	+	–	+	–	
	IHC	–	–	+	–	–	–	–	–	–	–	–	–	
Site	All sites	4	2	3	37	21	2	24	8	3	21	1	142	268
	Site 1	0	0	0	0	0	0	0	0	0	0	0	91	91
	Site 2	4	1	3	37	21	0	0	6	0	0	1	8	81
	Site 3	0	1	0	0	0	2	24	2	3	21	0	43	96

*Only positive counts of the test combinations are shown here (12/32). Test combinations that had no counts have not been included in the table (20/32). Table adapted from Jaramillo et al. (6)*.

### Bayesian Estimation of Diagnostic Sensitivity and Specificity

All models converged and showed no sign of autocorrelation with the exception of model 7. Model 7 showed bimodal posterior estimates; however, the model converged after restricting all DSp priors to be at least 0.5. Gelman-Rubin statistics of all model parameters were around 1. The posteriors estimates (median and 95% probability interval) of DSe, DSp and site prevalence based on the 3-populations models are presented in Table 6, and the results based on 2-populations models are presented in Table 7. Outputs from OpenBUGS including node statistics, density plots, plots of Gelman-Rubin diagnostic and plots of autocorrelation of all models are provided in the Supplementary Information.

**Table 6:** Posterior estimates for 3-populations models with 5-tests (Model 1), with 4-tests (Models 2 and 3), and with 3-tests (Model 4).

**Parameter**	**Model 1**		**Model 2**		**Model 3**		**Model 4**	
	**Median**	**95% probability interval**	**Median**	**95% probability interval**	**Median**	**95% probability interval**	**Median**	**95% probability interval**
**Prevalence**								
Site 1	1.70E-06	2.41E-07–5.52E-06	1.68E-05	2.48E-06–5.61E-05	1.67E-05	2.52E-06–5.55E-05	1.67E-05	2.34E-06–5.56E-05
Site 2	0.822	0.725–0.904	0.816	0.720–0.896	0.822	0.725–0.904	0.817	0.720–0.897
Site 3	0.010	0.0003–0.050	0.014	0.0007–0.060	0.010	0.0004–0.050	0.015	0.0007–0.061
**Test**								
PCR DSe	0.978	0.912–0.999	0.980	0.918–0.999	0.977	0.912–0.998	0.979	0.917–0.999
CELL DSe	0.950	0.856–0.996	0.956	0.861–0.997	0.949	0.855–0.949	0.955	0.858–0.997
NT DSe	0.085	0.034–0.168	n/a	n/a	0.086	0.034–0.168	n/a	n/a
HIST DSe	0.637	0.517–0.747	0.638	0.520–0.747	0.643	0.523–0.752	0.644	0.527–0.754
IHC DSe	0.051	0.015–0.118	0.051	0.015–0.119	n/a	n/a	n/a	n/a
PCR DSp	0.831	0.774–0.880	0.831	0.773–0.881	0.831	0.773–0.881	0.831	0.773–0.881
CELL DSp	0.993	0.972–0.999	0.994	0.975–0.999	0.993	0.973–0.999	0.994	0.975–0.999
NT DSp	0.744	0.680–0.801	n/a	n/a	0.744	0.679–0.801	n/a	n/a
HIST DSp	0.967	0.936–0.986	0.967	0.937–0.987	0.970	0.939–0.988	0.970	0.939–0.988
IHC DSp	0.996	0.981–0.999	0.996	0.982–0.999			n/a	n/a
Bayes *p*-value	0.036		0.157		0.007		0.038	

*DSe, Diagnostic test sensitivity; DSp, Diagnostic test specificity; n/a, not applicable*.

**Table 7:** Posterior estimates for 2-populations models with 5-tests: Model 5 for Site 1 and Site 2, Model 6 for Site 2 and Site 3, Model 7 for Site 1 and Site 3.

**Parameter**	**Model 5**		Model 6^*^		**Model 7**	
	**Median**	**95% probability interval**	**Median**	**95% probability interval**	**Median**	**95% probability interval**
**Prevalence**						
Site 1	1.69E-05	2.42E-06–5.64E-05	n/a	n/a	1.68E-05	2.43E-06–5.57E-05
Site 2	0.867	0.781–0.931	0.819	0.722–0.905	n/a	n/a
Site 3	n/a	n/a	0.008	0.0003–0.043	0.560	0.451–0.671
**Test**						
PCR DSe	0.976	0.915–0.998	0.973	0.898–0.998	0.525	0.393–0.659
CELL DSe	0.896	0.810–0.958	0.955	0.855–0.997	0.029	0.004–0.095
NT DSe	0.076	0.030–0.152	0.082	0.032–0.163	0.929	0.803–0.993
HIST DSe	0.608	0.497–0.716	0.641	0.522–0.750	0.109	0.045–0.205
IHC DSe	0.048	0.014–0.113	0.051	0.015–0.119	0.012	0.0004–0.063
PCR DSp	0.987	0.939–0.999	0.686	0.594–0.770	0.990	0.959–0.999
CELL DSp	0.991	0.957–0.999	0.984	0.947–0.998	0.993	0.969–0.999
NT DSp	0.993	0.963–0.999	0.546	0.502–0.628	0.992	0.959–0.999
HIST DSp	0.989	0.952–0.996	0.940	0.886–0.974	0.993	0.969–0.996
IHC DSp	0.991	0.959–0.999	0.993	0.967–0.993	0.993	0.969–0.999
Bayes *p*-value	0.481		0.237		0.823	

*DSe, diagnostic test sensitivity; DSp, diagnostic test specificity; n/a, not applicable*.

* *A converged model using a uniform (0.5,1) for all DSp prior distributions*.

**Figure 1:**
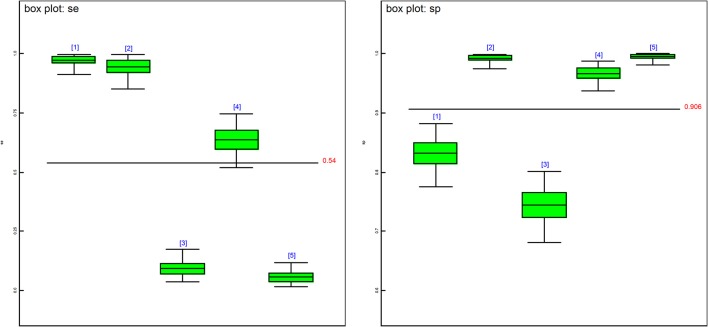
Boxplots of the posterior estimates based on model 1. se[1] = DSe of PCR, se[2] = DSe of CELL, se[3] = DSe of NT, se[4] = DSe of HIST, se[5] = DSe of IHC, sp[1] = DSp of PCR, sp[2] = DSp of CELL, sp[3] = DSe of NT, sp[4] = DSp of HIST, sp[5] = DSp of IHC.

The posterior estimates of site prevalence, DSe and DSp were very similar across all 3-populations models (Table 6). The posteriors estimates showed a very low probability of infection for Site 1, a high prevalence with a narrow probability interval for Site 2, and a low prevalence with a moderate probability interval for Site 3. In model 1, PCR had the highest DSe (0.978) followed by CELL (0.950) and HIST (0.637). Two tests, NT (0.085) and IHC (0.051), were not considered functional as their DSe estimates were very low. The DSe estimates of PCR and CELL had narrow probability intervals (0.912–0.999 and 0.856–0.996, respectively) giving a high certainty in the obtained estimates. In contrast, a larger uncertainty was observed for the DSe estimate of HIST (0.517–0.747). As for DSp, IHC (0.996) and CELL (0.993) yielded high DSp estimates with narrow probability intervals (0.981–0.999 and 0.972–0.999, respectively). HIST also had a high DSp estimate (0.967) with a slightly wider probability interval (0.936–0.986). PCR had a moderate DSp estimate (0.831) and NT (0.744) the lowest DSp estimate. DSe and DSp based on the models 2, 3, and 4 yielded consistent results with those obtained in model 1. Figure 1 shows boxplots of posterior estimates of all DSe and DSp based on model 1.

The covariance estimates between PCR and CELL and between HIST and IHC showed a very low test dependency between the pairs, where the highest covariance of the DSe estimate was 0.002 for the pair IHC and HIST and the highest covariance of the DSp estimate was 0.008 for the pair IHC and HIST.

For posterior estimates based on 2-populations models (Table 7), the model without site 3 (model 5) and the model without site 1 (model 6) had similar results compared to the 3-populations models (Table 6) regarding to site prevalence and DSp estimates. The model without site 2 (model 7), on the other hand, showed a considerable difference in DSe estimates. As for DSp, the model with 2-populations yielded very high DSp estimates, except for model 6 which had low estimates of DSp for PCR (0.686) and DSp for NT (0.546).

#### Difference in DSe and DSp Between Tests

For DSe, all pairs of the tests showed significant differences in their DSe estimates; except the pair of PCR and CELL and the pair of NT and IHC. The probability that PCR had a higher DSe estimate than CELL was 0.7913. The probability that NT had a higher DSe estimate than IHC was 0.7916. For DSp, the only non-significant difference was the pair of IHC and CELL. The probability that IHC had a higher DSp estimate than CELL was 0.649. A table containing the outputs from the Step function is available in the Supplementary Information.

#### Sensitivity Analysis

**Figure 2:**
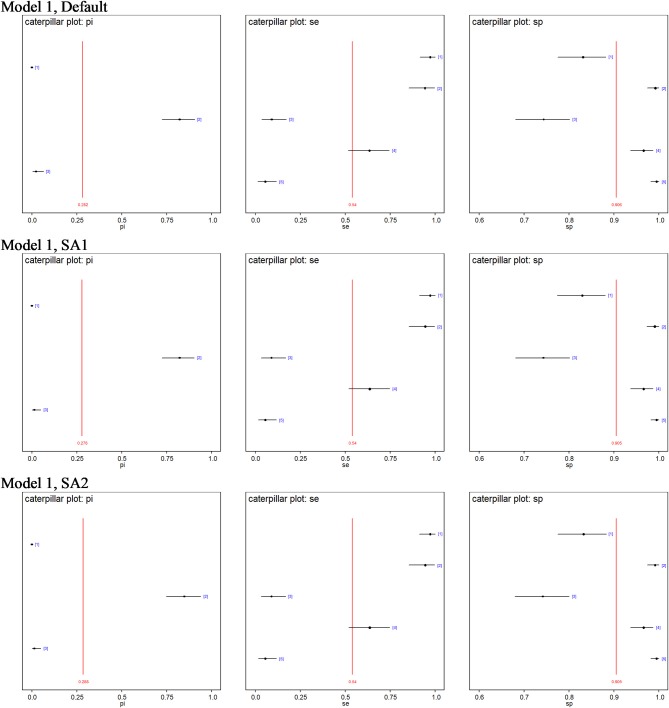
Caterpillar plots of the posterior estimates based on model 1. pi[1] = Site 1 prevalence, pi[2] = Site 2 prevalence, pi[3] = Site 3 prevalence, se[1] = DSe of PCR, se[2] = DSe of CELL, se[3] = DSe of NT, se[4] = DSe of HIST, se[5] = DSe of IHC, sp[1] = DSp of PCR, sp[2] = DSp of CELL, sp[3] = DSe of NT, sp[4] = DSp of HIST, sp[5] = DSp of IHC.

No site prevalence, DSe or DSp estimates were sensitive to changes in the priors of the models with 3-populations. Figure 2 shows caterpillar plots comparing the posterior estimates based on model 1, while the caterpillar plots based on models 2, 3, and 4 are available in the Supplementary Information.

## Discussion

This study evaluated the performance of five different diagnostic tests (real-time RT-PCR, CELL, histopathology, NT, and IHC) for detection of SAV infection and histopathological changes consistent with SAV infection in farmed Atlantic salmon, as listed in the chapter on infection with SAV in the OIE Manual of Diagnostic Tests for Aquatic Animals (20). All tests are direct, independent tests for the presence of SAV, with the exception of histopathology which detects tissue lesions as a sequela of SAV infection. In the current study the tests were applied to three different salmon farming sites, of which Site 1 was known to be free from SAV infection. Sites 2 and 3 were known to be infected by two different SAV genotypes and were assumed to be at different stages of infection. For the tests used, the SAV-genotype should not directly influence the test DSe and DSp although the infections themselves may behave differently in the infected populations, which may affect the test outcome.

When the tests were applied to a population considered free from SAV infection (Site 1) all tests performed well, and no sample was suggested SAV-positive by any of the test methods. When tests were applied to the infected populations, as expected, the test results from Site 2 and Site 3 showed that the two sites differed in their infection stage allowing us to investigate the diagnostic test performance using a Bayesian method. In our main model (model 1), all samples from the three different populations were compared to each other within the five-test panel. In this model, real-time RT-PCR was found to have the highest DSe. Real-time RT-PCR is commonly used as a screening test to determine the infection status of a population at on-growing seawater sites in salmonid aquaculture, and our results from this model support this as the method of choice for this purpose. Furthermore, isolation in cell culture was also found to have high DSe and DSp. While being more time-consuming and less suitable for screening purposes, an isolation in cell culture detects the presence of viable SAV-particles and not sections of genetic material only. A positive result indicates that the population may be active shedders of viral particles and thereby constitute a risk to naïve populations in the area. Models 2, 3, 4, 5, and 6 showed a close similarity in DSe to Model 1, with real-time RT-PCR having the highest DSe followed by isolation in cell culture and histopathology, respectively. SAV prevalence and DSp estimates are similar across the 3-populations models.

The consistent results for the posterior estimates across all the 3-populations models support the idea that these 3-populations models are more robust than the 2-populations models. Removing one population reduced the data by about 33%, which should significantly affect the estimates if the removed population provided a substantial contribution to the parameter estimates. When Site 1 was removed (model 6), the model became non-identifiable and required an informative prior for DSp. Similarly, when removing site 2 the model required informative priors for DSe. Our observation is in agreement with Berkvens et al. (32) and Dendukuri et al. (33) that adding constraints or using informative priors to some model parameters could provide a solution for a non-identifiable model.

The only previously published study on the DSe and DSp of real-time RT-PCR (head kidney) and cell culture (heart ventricle and heart kidney) for SAV were performed on samples from a Scottish outbreak of PD caused by SAV 1 (34). While the DSp estimates obtained were above 99% for both tests, the estimates for DSe were 50% (95% CI: 41–62%) for cell culture and 39% (95% CI: 31–49%) for real-time RT-PCR, respectively. This DSe are lower than those observed in the current study and could be due to the previous study not including heart tissue in the sampled material, an organ which should always be included when sampling for SAV detection according to the OIE manual (20). While not yet in common use in Norway, portable field kits for screening purposes are becoming available for a rapidly increasing range of infections agents in aquatic animals. A recent publication evaluated the DSe and DSp of a commercially available, portable field kit for SAV detection, which was found to have acceptable DSe and DSp although the reproducibility was somewhat reduced compared to the accredited in-laboratory method (35).

While none of the sites had a clinical outbreak of PD detectable by observation at the time of sampling, or prior to the time of sampling for Site 3, there were some important differences between the sites. Site 2 was sampled in the same month as the PD diagnosis was obtained, however as this area did not have compulsory screening for SAV the actual month of infection remains unknown. Site 3 was sampled 4 months after the diagnosis, however as this site was located in an area where compulsory screening was in place it is likely that the site was only infected shortly before the diagnosis was confirmed. While both experimental (16) and field (15) studies have shown differences between the two Norwegian SAV genotypes in the severity of the resultant clinical outbreaks, other biological factors influencing diagnostic test functionality appears to be relatively genotype-unspecific. A Norwegian experimental study found 57% of SAV2 cohabitant fish and 84% of SAV3 cohabitant fish to test positive by real-time RT-PCR at 2 weeks post-infection, which increased to 98% (regardless of SAV genotype) by week 3 and remained close to 100% until the end of the experiment (week 12) (16). Previous studies suggest that a prolonged positive PCR signal can be found in infected populations (36) and that populations remain positive until slaughter even when infected early during the seawater phase (24, 37). However, the prevalence of positive individuals decreased in some cases over time following the outbreak (24, 37). A recent Norwegian study sampling three Atlantic salmon populations at 2 weeks, 5, and 14 months after SAV2 detection, respectively, identified some individuals that tested positive for SAV2-RNA in red muscle tissue while the heart tissue tested negative (38). While scientific findings suggest that a PCR-positive population should test SAV-positive by real-time RT-PCR analyses at a population level at all times between infection and slaughter, there may be a reduced sensitivity at the individual fish level if not screening additional tissues later in the infection, perhaps particularly for SAV2 with its milder clinical course. It may be that a slower spread of SAV2 through the population, or the lack of tested muscle tissue for Site 3, accounts for the lower prevalence estimate obtained for this population despite it having been infected for several months.

The Norwegian experimental study of SAV2 and SAV3 found the onset of histopathological lesions from week 4 post-infection, with more than half of the fish still having SAV-related tissue changes as detected by histopathological examination at 12 weeks post-infection (16). Histopathological changes appear to be similar regardless of underlying SAV genotype (16, 17) and genotype should therefore be unlikely to affect the DSe and DSp of this test. The inherent biological variation in tissue lesions and the human factor of interpreting the range of possible changes likely account for the larger probability intervals obtained for the DSe and DSp estimates for this test method.

The NT is aimed at detecting antibodies/neutralizing activity against SAV in serum. High seroprevalences of virus neutralizing antibodies have been reported from studies of natural PD outbreaks (24, 37, 39), with the majority of populations remaining seropositive until slaughter (24, 37). No differences in virus titres or antibody levels were detected when comparing SAV2 and SAV3 experimental infections (16), suggesting that SAV-genotype differences are not the underlying reason for the observed result. The NT test performed particularly well for Site 3 (SAV2) which was sampled several months after diagnosis, and the failure to detect infectious virus particles by isolation in cell culture supports the interpretation that the population at this site was in a later stage of infection. It furthermore supports the assumption that Site 2 (SAV3) was infected relatively shortly before sampling, as research indicates that blood serum or plasma is suitable for a virus neutralization test that identifies neutralizing antibodies against SAV from ~3 weeks after SAV infection (26). It also highlights NT as a potential method for non-lethal screening of SAV exposure, which may be of particular relevance in the screening of larger, high-value salmon. However, the specificity of the NT test can be an issue, as cross-reactivity between PRV-1 and SAV has been demonstrated (40) In addition, vaccination against SAV, which is common in the endemic SAV3 area, will affect the results of the NT test. Successful vaccination against SAV will produce neutralizing antibodies of varying levels dependent on the vaccine type used, the efficacy of the vaccination and the time lag between vaccination and testing. In vaccinated fish, this test therefore has to be used with caution, and a positive result can only be interpreted as an indication of infection and not used as a confirmatory diagnostic test.

To our knowledge this is the first scientific publication of a field validation of the five major diagnostic tests recommended for SAV detection in accordance with the OIE Manual of Diagnostic Tests for Aquatic Animals (9). The OIE Manual lists recommended diagnostic methods for presumptive diagnosis, confirmatory diagnosis and targeted surveillance for SAV, and categorize the different methods based on availability, utility, and diagnostic sensitivity and specificity (20). While stating that these categorisations are somewhat subjective, the OIE concludes that the diagnostic tests classified as recommended methods and standard methods are acceptable due to their routine nature and the fact that they have been widely used without dubious results (20). For presumptive diagnosis histopathology and serum neutralization assay are listed as recommended methods while immunohistochemistry, real-time RT-PCR and RT-PCR with sequencing are listed as standard methods. For confirmatory diagnosis histopathology and RT-PCR with sequencing are recommended methods and immunohistochemistry, serum neutralization assay and real-time RT-PCR standard methods. For targeted surveillance real-time RT-PCR is listed as a standard method across fish life stages (fry, juveniles, and adults) (20). Field validation of diagnostic tests assesses the entire procedure from fish selection and sample collection to laboratory procedures, with each step potentially contributing to a reduction of the DSe and/or DSp. In our study, tissue samples included heart ventricle and mid-kidney, the recommended organs for the detection of SAV (2). The assessments of the real-time RT-PCR method for SAV were in accordance with that stipulated in the OIE manual (20), where it is listed as a standard method and is the method with highest ranking as a universal diagnostic test. Assessment of the analytical sensitivity (the limit of detection) and analytical specificity (the ability to detect the target sequence only) of the QnsP1 assay has shown it to be highly sensitive and specific with an ability to detect <0.1 TCID50 of virus stocks, with reproducible results at different RNA concentration levels (23), with a more recently developed assay reported to be 100 times more sensitive (41). Due to its application as a screening tool, and the potential consequences of false positive and false negative results, the DSe and DSp of real-time RT-PCR is of particular interest. A positive result (whether true or false) may be disputed by a range of stakeholders, in particular where the SAV-detection results in implementation of mitigation measures leading to economic losses and business interruption. While it was shown to perform well in the main model and in a recently infected population it showed a lower DSe in a population at a later stage of infection. A false negative result will be of particular importance in two scenarios: where the test-negative population sheds virus, or where the population is destined for export and subjected to import tests where SAV-RNA detection results in goods rejection and possibly subsequent trade sanctions.

In the case of a weak positive result, a follow-up test should be conducted in order to verify the presence of the infectious agent. In the case of SAV, isolation in cell culture, which was found to have high DSe and DSp in more recently infected populations, may be used to verify viral presence. Similarly, follow-up tests may be appropriate in cases where a negative PCR result is obtained from a population where the available epidemiological information suggest a high probability of infection. In this case, follow-up tests of serum samples may be appropriate in order to evaluate and monitor infection status. This is of particular relevance in cases where the consequences of false negative results are high, such as in cases of potential spread to previously non-infected areas or in relation to trade. Histopathological examination is an important test for identifying lesions associated with the agent of interest but also as a test for important differential diagnoses and passive surveillance. Based on our samples, IHC demonstrated both a limited DSe and DSp, which is as expected as the test is only recommended for samples from fish with acute necrosis of exocrine pancreatic tissue in the OIE manual (9).

Our results confirms that the evaluated tests ability correctly to detect SAV or the resultant disease is dependent on the infectious status of the population in question. To achieve the most reliable results for different situations such as early detection, monitoring prevalence and documentation of disease freedom, a thorough consideration of the appropriate method(s) has to be done at the beginning of the testing program.

## Data Availability Statement

The data sets supporting the conclusions of this study will be made available upon request to any qualified researcher.

## Ethics Statement

Ethical review and approval was not required for the animal study because this study was performed in accordance with the ethical guidelines of the Norwegian Veterinary Institute. All fish were sedated and humanely euthanized prior to sample collection in accordance with standard procedures. Written informed consent was obtained from the owners for the participation of their animals in this study.

## Author Contributions

All authors contributed to ideas for analysis, discussion of methodology and results, writing and editing of the manuscript.

### Conflict of Interest

The authors declare that the research was conducted in the absence of any commercial or financial relationships that could be construed as a potential conflict of interest.
