# Supplementary material for: Field Evaluation of Diagnostic Test Sensitivity and Specificity for Salmonid Alphavirus (SAV) Infection and Pancreas Disease (PD) in Farmed Atlantic salmon (Salmo salar L.) in Norway Using Bayesian Latent Class Analysis
Source: Front Vet Sci. 2019 Nov 28;6:419. doi: 10.3389/fvets.2019.00419 (PMC6893554; doi:10.3389/fvets.2019.00419)

Supplementary information

#R Code - Plotting prior distributions of site prevalence and DSe, DSp

```
par(mfrow=c(2,2))
plot(density(rbeta(1000000,2,100000)), main="Prior distribution of Site1 prevalence")
plot(density(rbeta(1000000,6,4)), main="Prior distribution of Site2 prevalence")
plot(density(rbeta(1000000,1,1)), main="Prior distribution of Site3 prevalence")
plot(density(rbeta(1000000,1,1)), main="Prior distribution of DSe, DSp for all 5 tests")
```

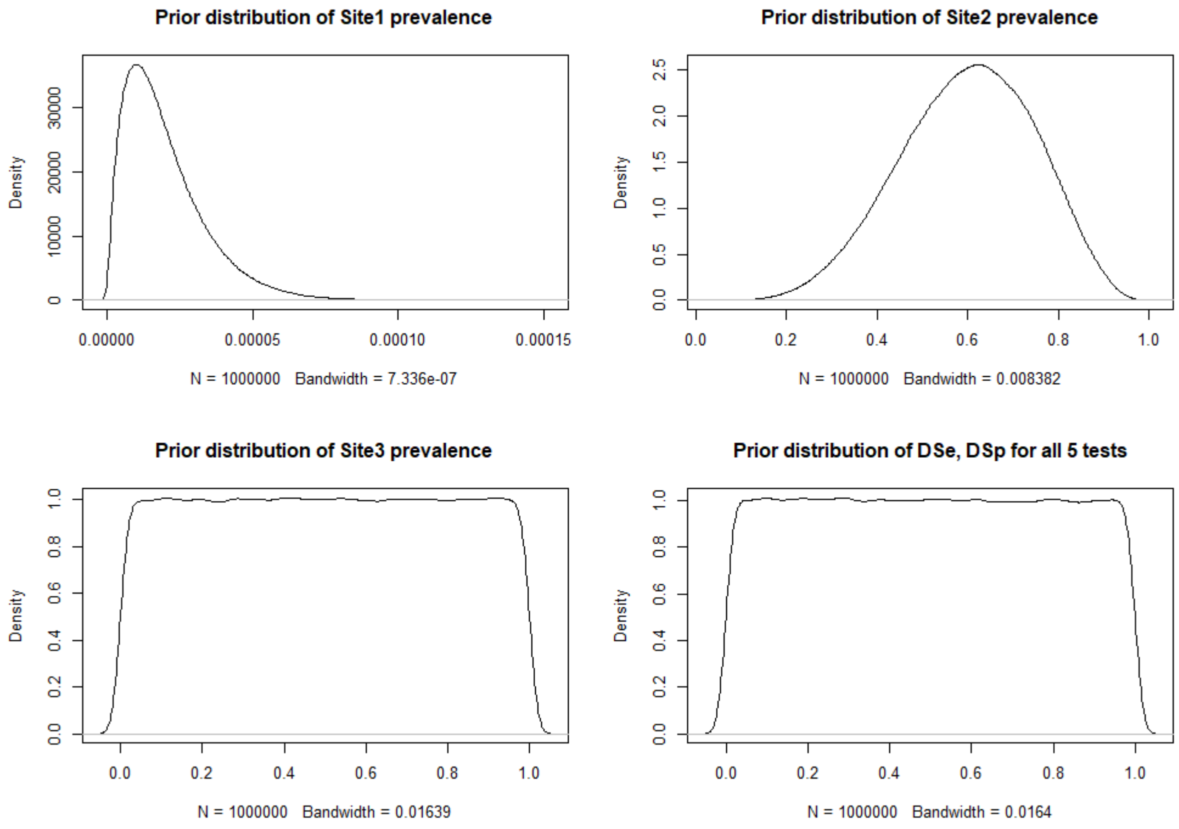

#R Code - Convergence diagnostics

```
library(coda)
#import the chains
chain1 = read.coda("./CODAchain1_model1.txt", "./CODAindex_model1.txt")
chain2 = read.coda("./CODAchain2_model1.txt", "./CODAindex_model1.txt")
chain3 = read.coda("./CODAchain3_model1.txt", "./CODAindex_model1.txt")

mh.list = mcmc.list(list(chain1, chain2, chain3))

#Traceplot
plot(mh.list, trace = T, density = T, auto.layout = T)

#Gelman-Rubin diagnostics
gelman.diag(mh.list)
```

#OpenBUGS code – An example of model 1 code

```
5 tests 3 populations
model
{
# multinomial models where k refers to population, and K refers to the number of populations
for (k in 1:K){
y[k,1:2,1:2,1:2,1:2,1:2] ~ dmulti(p[k,1:2,1:2,1:2,1:2,1:2], n[k])
}
# cell probabilities expressed in terms of se (sensitivity), sp (specificity), pi(prevalence), cov
(covariance)
for (k in 1:K){
p[k,1,1,1,1,1] <- pi[k]*(se[1]*se[2]+cov12[1])*se[3]*(se[4]*se[5]+cov45[1]) + (1-pi[k])*((1-sp[1])*(1-
sp[2])+cov12[2])*(1-sp[3])*((1-sp[4])*(1-sp[5])+cov45[2])
p[k,1,1,1,1,2] <- pi[k]*(se[1]*se[2]+cov12[1])*se[3]*(se[4]*(1-se[5])-cov45[1]) + (1-pi[k])*((1-
sp[1])*(1-
sp[2])+cov12[2])*(1-sp[3])*((1-sp[4])*sp[5]-cov45[2])
p[k,1,1,1,2,1] <- pi[k]*(se[1]*se[2]+cov12[1])*se[3]*((1-se[4])*se[5]-cov45[1]) + (1-pi[k])*((1-
sp[1])*(1-
sp[2])+cov12[2])*(1-sp[3])*(sp[4]*(1-sp[5])-cov45[2])
p[k,1,1,1,2,2] <- pi[k]*(se[1]*se[2]+cov12[1])*se[3]*((1-se[4])*(1-se[5])+cov45[1]) + (1-pi[k])*((1-
sp[1])*(1-sp[2])+cov12[2])*(1-sp[3])*(sp[4]*sp[5]+cov45[2])
p[k,1,1,2,1,1] <- pi[k]*(se[1]*se[2]+cov12[1])*(1-se[3])*(se[4]*se[5]+cov45[1]) + (1-pi[k])*((1-
sp[1])*(1-
sp[2])+cov12[2])*sp[3]*((1-sp[4])*(1-sp[5])+cov45[2])
p[k,1,1,2,1,2] <- pi[k]*(se[1]*se[2]+cov12[1])*(1-se[3])*(se[4]*(1-se[5])-cov45[1]) + (1-pi[k])*((1-
sp[1])*(1-sp[2])+cov12[2])*sp[3]*((1-sp[4])*sp[5]-cov45[2])
p[k,1,1,2,2,1] <- pi[k]*(se[1]*se[2]+cov12[1])*(1-se[3])*((1-se[4])*se[5]-cov45[1]) + (1-pi[k])*((1-
sp[1])*(1-sp[2])+cov12[2])*sp[3]*(sp[4]*(1-sp[5])-cov45[2])
p[k,1,1,2,2,2] <- pi[k]*(se[1]*se[2]+cov12[1])*(1-se[3])*((1-se[4])*(1-se[5])+cov45[1]) + (1-pi[k])*((1-
sp[1])*(1-sp[2])+cov12[2])*sp[3]*(sp[4]*sp[5]+cov45[2])
p[k,1,2,1,1,1] <- pi[k]*(se[1]*(1-se[2])-cov12[1])*se[3]*(se[4]*se[5]+cov45[1]) + (1-pi[k])*((1-
sp[1])*sp[2]-cov12[2])*(1-sp[3])*((1-sp[4])*(1-sp[5])+cov45[2])
p[k,1,2,1,1,2] <- pi[k]*(se[1]*(1-se[2])-cov12[1])*se[3]*(se[4]*(1-se[5])-cov45[1]) + (1-pi[k])*((1-
sp[1])*sp[2]-cov12[2])*(1-sp[3])*((1-sp[4])*sp[5]-cov45[2])
p[k,1,2,1,2,1] <- pi[k]*(se[1]*(1-se[2])-cov12[1])*se[3]*((1-se[4])*se[5]-cov45[1]) + (1-pi[k])*((1-
sp[1])*sp[2]-cov12[2])*(1-sp[3])*(sp[4]*(1-sp[5])-cov45[2])
p[k,1,2,1,2,2] <- pi[k]*(se[1]*(1-se[2])-cov12[1])*se[3]*((1-se[4])*(1-se[5])+cov45[1]) + (1-pi[k])*((1-
sp[1])*sp[2]-cov12[2])*(1-sp[3])*(sp[4]*sp[5]+cov45[2])
p[k,1,2,2,1,1] <- pi[k]*(se[1]*(1-se[2])-cov12[1])*(1-se[3])*(se[4]*se[5]+cov45[1]) + (1-pi[k])*((1-
sp[1])*sp[2]-cov12[2])*sp[3]*((1-sp[4])*(1-sp[5])+cov45[2])
p[k,1,2,2,1,2] <- pi[k]*(se[1]*(1-se[2])-cov12[1])*(1-se[3])*(se[4]*(1-se[5])-cov45[1]) + (1-pi[k])*((1-
sp[1])*sp[2]-cov12[2])*sp[3]*((1-sp[4])*sp[5]-cov45[2])
p[k,1,2,2,2,1] <- pi[k]*(se[1]*(1-se[2])-cov12[1])*(1-se[3])*((1-se[4])*se[5]-cov45[1]) + (1-pi[k])*((1-
sp[1])*sp[2]-cov12[2])*sp[3]*(sp[4]*(1-sp[5])-cov45[2])
p[k,1,2,2,2,2] <- pi[k]*(se[1]*(1-se[2])-cov12[1])*(1-se[3])*((1-se[4])*(1-se[5])+cov45[1]) + (1-
pi[k])*((1-
sp[1])*sp[2]-cov12[2])*sp[3]*(sp[4]*sp[5]+cov45[2])
p[k,2,1,1,1,1] <- pi[k]*((1-se[1])*se[2]-cov12[1])*se[3]*(se[4]*se[5]+cov45[1]) + (1-pi[k])*(sp[1]*(1-
sp[2])-cov12[2])*(1-sp[3])*((1-sp[4])*(1-sp[5])+cov45[2])
p[k,2,1,1,1,2] <- pi[k]*((1-se[1])*se[2]-cov12[1])*se[3]*(se[4]*(1-se[5])-cov45[1]) + (1-
pi[k])*(sp[1]*(1-
```

```
sp[2])-cov12[2])*(1-sp[3])*((1-sp[4])*sp[5]-cov45[2])
p[k,2,1,1,2,1] <- pi[k]*((1-se[1])*se[2]-cov12[1])*se[3]*((1-se[4])*se[5]-cov45[1]) + (1-
pi[k])*(sp[1]*(1-
sp[2])-cov12[2])*(1-sp[3])*(sp[4]*(1-sp[5])-cov45[2])
p[k,2,1,1,2,2] <- pi[k]*((1-se[1])*se[2]-cov12[1])*se[3]*((1-se[4])*(1-se[5])+cov45[1]) + (1-
pi[k])*(sp[1]*(1-sp[2])-cov12[2])*(1-sp[3])*(sp[4]*sp[5]+cov45[2])
p[k,2,1,2,1,1] <- pi[k]*((1-se[1])*se[2]-cov12[1])*(1-se[3])*(se[4]*se[5]+cov45[1]) + (1-
pi[k])*(sp[1]*(1-
sp[2])-cov12[2])*sp[3]*((1-sp[4])*(1-sp[5])+cov45[2])
p[k,2,1,2,1,2] <- pi[k]*((1-se[1])*se[2]-cov12[1])*(1-se[3])*(se[4]*(1-se[5])-cov45[1]) + (1-
pi[k])*(sp[1]*(1-sp[2])-cov12[2])*sp[3]*((1-sp[4])*sp[5]-cov45[2])
p[k,2,1,2,2,1] <- pi[k]*((1-se[1])*se[2]-cov12[1])*(1-se[3])*((1-se[4])*se[5]-cov45[1]) + (1-
pi[k])*(sp[1]*(1-sp[2])-cov12[2])*sp[3]*(sp[4]*(1-sp[5])-cov45[2])
p[k,2,1,2,2,2] <- pi[k]*((1-se[1])*se[2]-cov12[1])*(1-se[3])*((1-se[4])*(1-se[5])+cov45[1]) + (1-
pi[k])*(sp[1]*(1-sp[2])-cov12[2])*sp[3]*(sp[4]*sp[5]+cov45[2])
p[k,2,2,1,1,1] <- pi[k]*((1-se[1])*(1-se[2])+cov12[1])*se[3]*(se[4]*se[5]+cov45[1]) + (1-
pi[k])*(sp[1]*sp[2]+cov12[2])*(1-sp[3])*((1-sp[4])*(1-sp[5])+cov45[2])
p[k,2,2,1,1,2] <- pi[k]*((1-se[1])*(1-se[2])+cov12[1])*se[3]*(se[4]*(1-se[5])-cov45[1]) + (1-
pi[k])*(sp[1]*sp[2]+cov12[2])*(1-sp[3])*((1-sp[4])*sp[5]-cov45[2])
p[k,2,2,1,2,1] <- pi[k]*((1-se[1])*(1-se[2])+cov12[1])*se[3]*((1-se[4])*se[5]-cov45[1]) + (1-
pi[k])*(sp[1]*sp[2]+cov12[2])*(1-sp[3])*(sp[4]*(1-sp[5])-cov45[2])
p[k,2,2,1,2,2] <- pi[k]*((1-se[1])*(1-se[2])+cov12[1])*se[3]*((1-se[4])*(1-se[5])+cov45[1]) + (1-
pi[k])*(sp[1]*sp[2]+cov12[2])*(1-sp[3])*(sp[4]*sp[5]+cov45[2])
p[k,2,2,2,1,1] <- pi[k]*((1-se[1])*(1-se[2])+cov12[1])*(1-se[3])*(se[4]*se[5]+cov45[1]) + (1-
pi[k])*(sp[1]*sp[2]+cov12[2])*sp[3]*((1-sp[4])*(1-sp[5])+cov45[2])
p[k,2,2,2,1,2] <- pi[k]*((1-se[1])*(1-se[2])+cov12[1])*(1-se[3])*(se[4]*(1-se[5])-cov45[1]) + (1-
pi[k])*(sp[1]*sp[2]+cov12[2])*sp[3]*((1-sp[4])*sp[5]-cov45[2])
p[k,2,2,2,2,1] <- pi[k]*((1-se[1])*(1-se[2])+cov12[1])*(1-se[3])*((1-se[4])*se[5]-cov45[1]) + (1-
pi[k])*(sp[1]*sp[2]+cov12[2])*sp[3]*(sp[4]*(1-sp[5])-cov45[2])
p[k,2,2,2,2,2] <- pi[k]*((1-se[1])*(1-se[2])+cov12[1])*(1-se[3])*((1-se[4])*(1-se[5])+cov45[1]) + (1-
pi[k])*(sp[1]*sp[2]+cov12[2])*sp[3]*(sp[4]*sp[5]+cov45[2])
}
# prior distributions
se[1] ~ dbeta(1,1)
sp[1] ~ dbeta(1,1)
se[2] ~ dbeta(1,1)
sp[2] ~ dbeta(1,1)
se[3] ~ dbeta(1,1)
sp[3] ~ dbeta(1,1)
se[4] ~ dbeta(1,1)
sp[4] ~ dbeta(1,1)
se[5] ~ dbeta(1,1)
sp[5] ~ dbeta(1,1)
#sum of probabilities
test1 <-
p[2,1,1,1,1,1]+p[2,1,1,1,1,2]+p[2,1,1,1,2,1]+p[2,1,1,1,2,2]+p[2,1,1,2,1,1]+p[2,1,1,2,1,2]+p[2,1,1,2,2,1]
+
p[2,1,1,2,2,2]+p[2,1,2,1,1,1]+p[2,1,2,1,1,2]+p[2,1,2,1,2,1]+p
[2,1,2,1,2,2]+p[2,1,2,2,1,1]+p[2,1,2,2,1,2]+p[2,1,2,2,2,1]+p[2,1,2,2,2,2]+p[2,2,1,1,1,1]+p[2,2,1,1,1,2]+
p
[2,2,1,1,2,1]+p[2,2,1,1,2,2]+p[2,2,1,2,1,1]+p[2,2,1,2,1,2]+p
```

```
[2,2,1,2,2,1]+p[2,2,1,2,2,2]+p[2,2,2,1,1,1]+p[2,2,2,1,1,2]+p[2,2,2,1,2,1]+p[2,2,2,1,2,2]+p[2,2,2,2,1,1]+
p
[2,2,2,2,1,2]+p[2,2,2,2,2,1]+p[2,2,2,2,2,2]

for (k in 1:1){
pi[k]~dbeta(2,1000000)
}

for(k in 2:2){
pi[k]~dbeta(6,4)
}

for(k in 3:3){
pi[k]~dbeta(1,1)
}

# Covariance note: cov12[1] ~ sensitivity, cov12[2] ~ specificity
cov12_low[1] <- max(-(1-se[1])*(1-se[2]),-se[1]*se[2])
cov12_upp[1] <- min(se[1]*(1-se[2]),(1-se[1])*se[2])
cov12_low[2] <- max(-(1-sp[1])*(1-sp[2]),-sp[1]*sp[2])
cov12_upp[2] <- min(sp[1]*(1-sp[2]),(1-sp[1])*sp[2])
cov12[1] ~ dunif(cov12_low[1],cov12_upp[1])
cov12[2] ~ dunif(cov12_low[2],cov12_upp[2])
cov45_low[1] <- max(-(1-se[4])*(1-se[5]),-se[4]*se[5])
cov45_upp[1] <- min(se[4]*(1-se[5]),(1-se[4])*se[5])
cov45_low[2] <- max(-(1-sp[4])*(1-sp[5]),-sp[4]*sp[5])
cov45_upp[2] <- min(sp[4]*(1-sp[5]),(1-sp[4])*sp[5])
cov45[1] ~ dunif(cov45_low[1],cov45_upp[1])
cov45[2] ~ dunif(cov45_low[2],cov45_upp[2])

#difference in Se and Sp between tests
sediff[1] <- se[1]- se[2]
sediff[2] <- se[1]- se[3]
sediff[3] <- se[1]- se[4]
sediff[4] <- se[1]- se[5]
sediff[5] <- se[2]- se[1]
sediff[6] <- se[2]- se[3]
sediff[7] <- se[2]- se[4]
sediff[8] <- se[2]- se[5]
sediff[9] <- se[3]- se[1]
sediff[10] <- se[3]- se[2]
sediff[11] <- se[3]- se[4]
sediff[12] <- se[3]- se[5]
sediff[13] <- se[4]- se[1]
sediff[14] <- se[4]- se[2]
sediff[15] <- se[4]- se[3]
sediff[16] <- se[4]- se[5]
sediff[17] <- se[5]- se[1]
sediff[18] <- se[5]- se[2]
sediff[19] <- se[5]- se[3]
sediff[20] <- se[5]- se[4]
spdiff[1] <- sp[1]- sp[2]
```

```
spdiff[2] <- sp[1]- sp[3]
spdiff[3] <- sp[1]- sp[4]
spdiff[4] <- sp[1]- sp[5]
spdiff[5] <- sp[2]- sp[1]
spdiff[6] <- sp[2]- sp[3]
spdiff[7] <- sp[2]- sp[4]
spdiff[8] <- sp[2]- sp[5]
spdiff[9] <- sp[3]- sp[1]
spdiff[10] <- sp[3]- sp[2]
spdiff[11] <- sp[3]- sp[4]
spdiff[12] <- sp[3]- sp[5]
spdiff[13] <- sp[4]- sp[1]
spdiff[14] <- sp[4]- sp[2]
spdiff[15] <- sp[4]- sp[3]
spdiff[16] <- sp[4]- sp[5]
spdiff[17] <- sp[5]- sp[1]
spdiff[18] <- sp[5]- sp[2]
spdiff[19] <- sp[5]- sp[3]
spdiff[20] <- sp[5]- sp[4]
#Step function analysis
Prsediff[1] <- step(sediff[1])
Prsediff[2] <- step(sediff[2])
Prsediff[3] <- step(sediff[3])
Prsediff[4] <- step(sediff[4])
Prsediff[5] <- step(sediff[5])
Prsediff[6] <- step(sediff[6])
Prsediff[7] <- step(sediff[7])
Prsediff[8] <- step(sediff[8])
Prsediff[9] <- step(sediff[9])
Prsediff[10] <- step(sediff[10])
Prsediff[11] <- step(sediff[11])
Prsediff[12] <- step(sediff[12])
Prsediff[13] <- step(sediff[13])
Prsediff[14] <- step(sediff[14])
Prsediff[15] <- step(sediff[15])
Prsediff[16] <- step(sediff[16])
Prsediff[17] <- step(sediff[17])
Prsediff[18] <- step(sediff[18])
Prsediff[19] <- step(sediff[19])
Prsediff[20] <- step(sediff[20])
Prspdiff[1] <- step(spdiff[1])
Prspdiff[2] <- step(spdiff[2])
Prspdiff[3] <- step(spdiff[3])
Prspdiff[4] <- step(spdiff[4])
Prspdiff[5] <- step(spdiff[5])
Prspdiff[6] <- step(spdiff[6])
Prspdiff[7] <- step(spdiff[7])
Prspdiff[8] <- step(spdiff[8])
Prspdiff[9] <- step(spdiff[9])
Prspdiff[10] <- step(spdiff[10])
Prspdiff[11] <- step(spdiff[11])
Prspdiff[12] <- step(spdiff[12])
```

```
Prspdiff[13] <- step(spdiff[13])
Prspdiff[14] <- step(spdiff[14])
Prspdiff[15] <- step(spdiff[15])
Prspdiff[16] <- step(spdiff[16])
Prspdiff[17] <- step(spdiff[17])
Prspdiff[18] <- step(spdiff[18])
Prspdiff[19] <- step(spdiff[19])
Prspdiff[20] <- step(spdiff[20])

# code for Bayesian p-value
for (k in 1:K){
s[k,1:2,1:2,1:2,1:2,1:2] ~ dmulti(p[k,1:2,1:2,1:2,1:2,1:2],n[k])
for (i1 in 1:2){
for (i2 in 1:2){
for (i3 in 1:2){
for (i4 in 1:2){
for (i5 in 1:2){
x2[k,i1,i2,i3,i4,i5] <- pow(y[k,i1,i2,i3,i4,i5]-p[k,i1,i2,i3,i4,i5]*n[k],2)/(p[k,i1,i2,i3,i4,i5]*n[k])
ref[k,i1,i2,i3,i4,i5] <- pow(s[k,i1,i2,i3,i4,i5]-p[k,i1,i2,i3,i4,i5]*n[k],2)/(p[k,i1,i2,i3,i4,i5]*n[k])
}}}}}}
x2total <- sum(x2[,,,,,])
reftotal <- sum(ref[,,,,,])
bayesp <- step(reftotal-x2total)
}
}
```

MCMC results of 7 models

1. A model with 3 populations (Site 1, Site 2, Site 3) and 5 tests (PCR, CELL, NT, HIST, IHC) – ***This is the model 1 in the main text.***

1.1. Priors of model 1

**Parameters**	**Prior distribution**
Site 1 prevalence	beta(2,100000)
Site 2 prevalence	beta(6,4)
Site 3 prevalence	beta(1,1)
DSe of all tests	beta(1,1)
DSp of all tests	beta(1,1)

1.2. MCMC Results

1.2.1.Node statistics of model 1

model	population	test	parameters	mean	sd	MC_error	val2.5pc	median	val97.5pc	start	sample
1	1,2,3	PCR, CELL, NT, HIST, IHC	bayesp	0.03604	1.86E-01	0.001155	0	0	1	10000	27000
1	1,2,3	PCR, CELL, NT, HIST, IHC	pi[1]	2.00E-06	1.40E-06	8.78E-09	2.41E-07	1.70E-06	5.52E-06	10000	27000
1	1,2,3	PCR, CELL, NT, HIST, IHC	pi[2]	0.8205	4.59E-02	2.83E-04	0.725	0.8225	0.9049	10000	27000
1	1,2,3	PCR, CELL, NT, HIST, IHC	pi[3]	0.01436	1.35E-02	8.46E-05	3.77E-04	0.01047	0.05036	10000	27000
1	1,2,3	PCR, CELL, NT, HIST, IHC	se[1]	0.9721	2.33E-02	2.24E-04	0.9127	0.9782	0.999	10000	27000
1	1,2,3	PCR, CELL, NT, HIST, IHC	se[2]	0.9439	3.78E-02	2.64E-04	0.8563	0.9501	0.9962	10000	27000
1	1,2,3	PCR, CELL, NT, HIST, IHC	se[3]	0.08989	3.47E-02	2.05E-04	0.03403	0.08577	0.1682	10000	27000
1	1,2,3	PCR, CELL, NT, HIST, IHC	se[4]	0.6361	5.85E-02	3.54E-04	0.5175	0.6376	0.7475	10000	27000
1	1,2,3	PCR, CELL, NT, HIST, IHC	se[5]	0.05559	2.68E-02	1.63E-04	0.01582	0.05138	0.118	10000	27000
1	1,2,3	PCR, CELL, NT, HIST, IHC	sp[1]	0.8303	2.72E-02	1.72E-04	0.7742	0.8314	0.8805	10000	27000
1	1,2,3	PCR, CELL, NT, HIST, IHC	sp[2]	0.9913	0.007105	6.26E-05	0.9729	0.993	0.9996	10000	27000
1	1,2,3	PCR, CELL, NT, HIST, IHC	sp[3]	0.7432	0.03085	1.87E-04	0.6806	0.744	0.8013	10000	27000
1	1,2,3	PCR, CELL, NT, HIST, IHC	sp[4]	0.9659	0.01296	8.20E-05	0.9365	0.9675	0.9864	10000	27000
1	1,2,3	PCR, CELL, NT, HIST, IHC	sp[5]	0.9951	0.004922	3.83E-05	0.9819	0.9966	0.9999	10000	27000

Note: pi[1] = Site 1 prevalence, pi[2] = Site 2 prevalence, pi[3] = Site 3 prevalence, se[1] = DSe of PCR, se[2] = DSe of CELL, se[3] = DSe of NT, se[4] = DSe of HIST, se[5] = DSe of IHC, sp[1] = DSp of PCR, sp[2] = DSp of CELL, sp[3] = DSp of NT, sp[4] = DSp of HIST, sp[5] = DSp of IHC

1.2.2.Density plots of posterior distributions of model 1

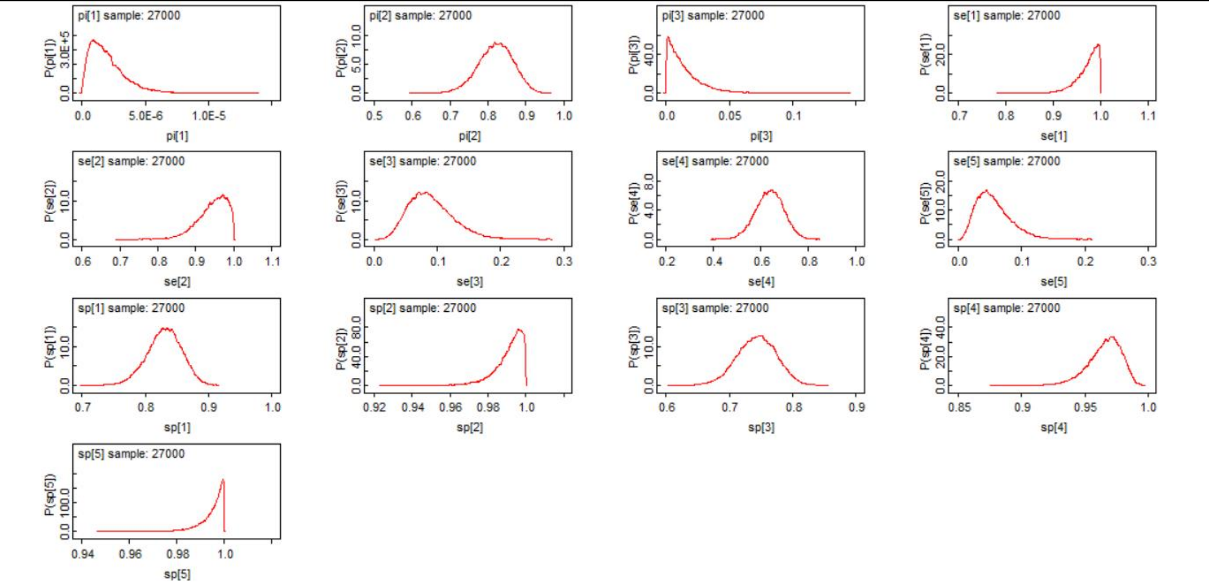

Note: pi[1] = Site 1 prevalence, pi[2] = Site 2 prevalence, pi[3] = Site 3 prevalence, se[1] = DSe of PCR, se[2] = DSe of CELL, se[3] = DSe of NT, se[4] = DSe of HIST, se[5] = DSe of IHC, sp[1] = DSp of PCR, sp[2] = DSp of CELL, sp[3] = DSp of NT, sp[4] = DSp of HIST, sp[5] = DSp of IHC

1.2.3 Plots of Gelman-Rubin diagnostic of model 1

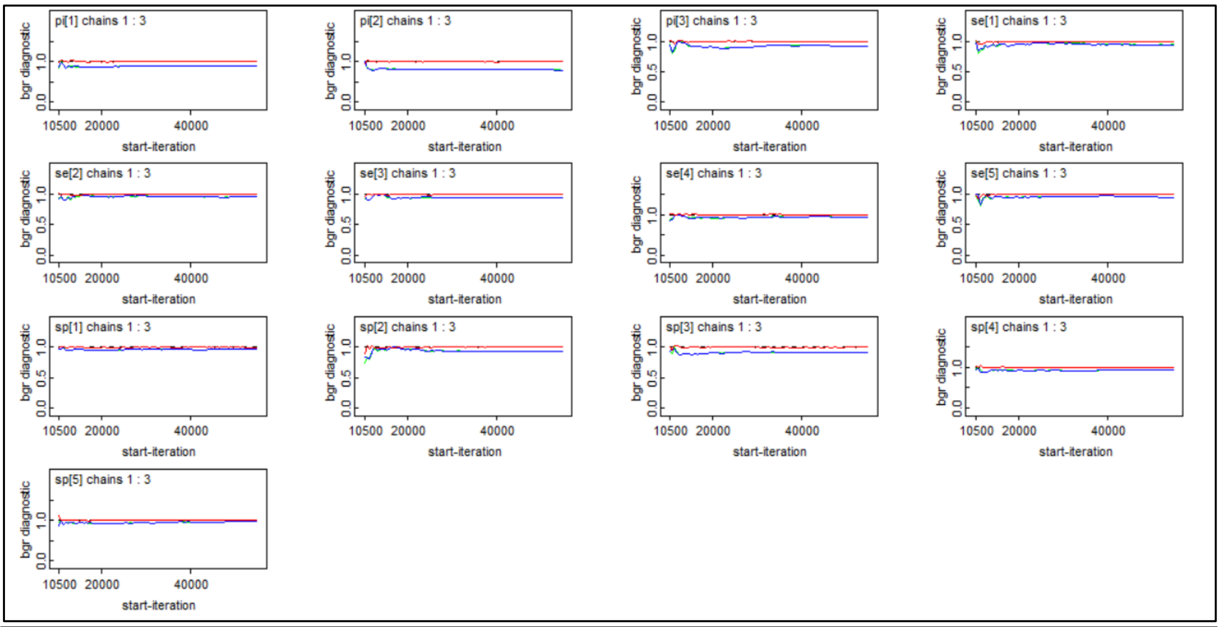

Note: pi[1] = Site 1 prevalence, pi[2] = Site 2 prevalence, pi[3] = Site 3 prevalence, se[1] = DSe of PCR, se[2] = DSe of CELL, se[3] = DSe of NT, se[4] = DSe of HIST, se[5] = DSe of IHC, sp[1] = DSp of PCR, sp[2] = DSp of CELL, sp[3] = DSp of NT, sp[4] = DSp of HIST, sp[5] = DSp of IHC

1.2.5 Plots of Gelman-Rubin diagnostic of model 1

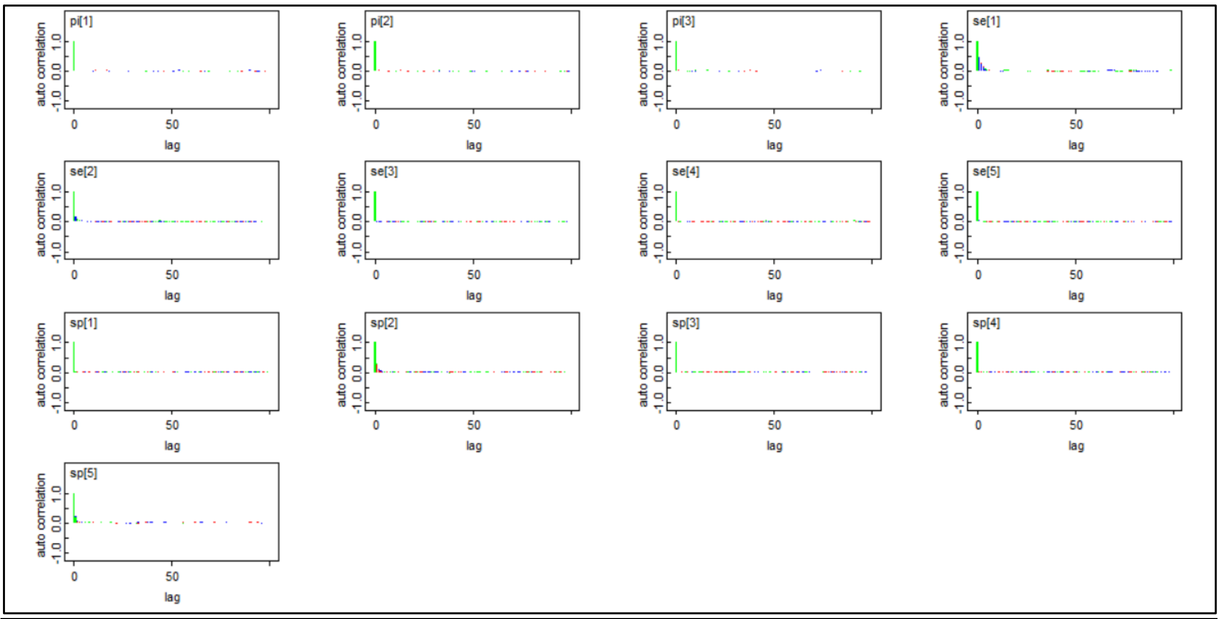

Note: pi[1] = Site 1 prevalence, pi[2] = Site 2 prevalence, pi[3] = Site 3 prevalence, se[1] = DSe of PCR, se[2] = DSe of CELL, se[3] = DSe of NT, se[4] = DSe of HIST, se[5] = DSe of IHC, sp[1] = DSp of PCR, sp[2] = DSp of CELL, sp[3] = DSp of NT, sp[4] = DSp of HIST, sp[5] = DSp of IHC

2. A model with 3 populations (Site 1, Site 2, Site 3) and 4 tests (PCR, CELL, HIST, IHC)

2.1. Priors of model 2

**Parameters**	**Prior distribution**
Site 1 prevalence	beta(2,100000)
Site 2 prevalence	beta(6,4)
Site 3 prevalence	beta(1,1)
DSe of all tests	beta(1,1)
DSe of all tests	beta(1,1)

2.2. MCMC Results

2.2.1.Node statistics of model 2

model	populati	test	paramete	mean	sd	MC_err	val2.5p	median	val97.5	start	sample
2	1,2,3	PCR, CELL, HIST, IHC	bayesp	0.1577	0.3644	0.002267	0	0	1	10000	27000
2	1,2,3	PCR, CELL, HIST, IHC	pi[1]	2.01E-05	1.42E-05	9.12E-08	2.48E-06	1.68E-05	5.61E-05	10000	27000
2	1,2,3	PCR, CELL, HIST, IHC	pi[2]	0.8143	0.04492	2.85E-04	0.7203	0.8164	0.8966	10000	27000
2	1,2,3	PCR, CELL, HIST, IHC	pi[3]	0.01895	0.01605	9.72E-05	7.18E-04	0.01487	0.06049	10000	27000
2	1,2,3	PCR, CELL, HIST, IHC	se[1]	0.9745	0.02206	2.15E-04	0.9182	0.9804	0.9992	10000	27000
2	1,2,3	PCR, CELL, HIST, IHC	se[2]	0.9492	0.03654	2.74E-04	0.8618	0.9564	0.9972	10000	27000
2	1,2,3	PCR, CELL, HIST, IHC	se[4]	0.6381	0.05807	3.57E-04	0.5205	0.6389	0.7471	10000	27000
2	1,2,3	PCR, CELL, HIST, IHC	se[5]	0.05568	0.02704	1.66E-04	0.01557	0.05159	0.1198	10000	27000
2	1,2,3	PCR, CELL, HIST, IHC	sp[1]	0.8306	0.02736	1.71E-04	0.7739	0.8316	0.8815	10000	27000
2	1,2,3	PCR, CELL, HIST, IHC	sp[2]	0.9928	0.006639	5.32E-05	0.9754	0.9946	0.9998	10000	27000
2	1,2,3	PCR, CELL, HIST, IHC	sp[4]	0.9664	0.01301	8.28E-05	0.9371	0.9679	0.9872	10000	27000
2	1,2,3	PCR, CELL, HIST, IHC	sp[5]	0.9951	0.004881	3.63E-05	0.982	0.9966	0.9999	10000	27000

Note: pi[1] = Site 1 prevalence, pi[2] = Site 2 prevalence, pi[3] = Site 3 prevalence, se[1] = DSe of PCR, se[2] = DSe of CELL, se[4] = DSe of HIST, se[5] = DSe of IHC, sp[1] = DSp of PCR, sp[2] = DSp of CELL, sp[4] = DSp of HIST, sp[5] = DSp of IHC

2.2.2.Density plots of posterior distributions of model 2

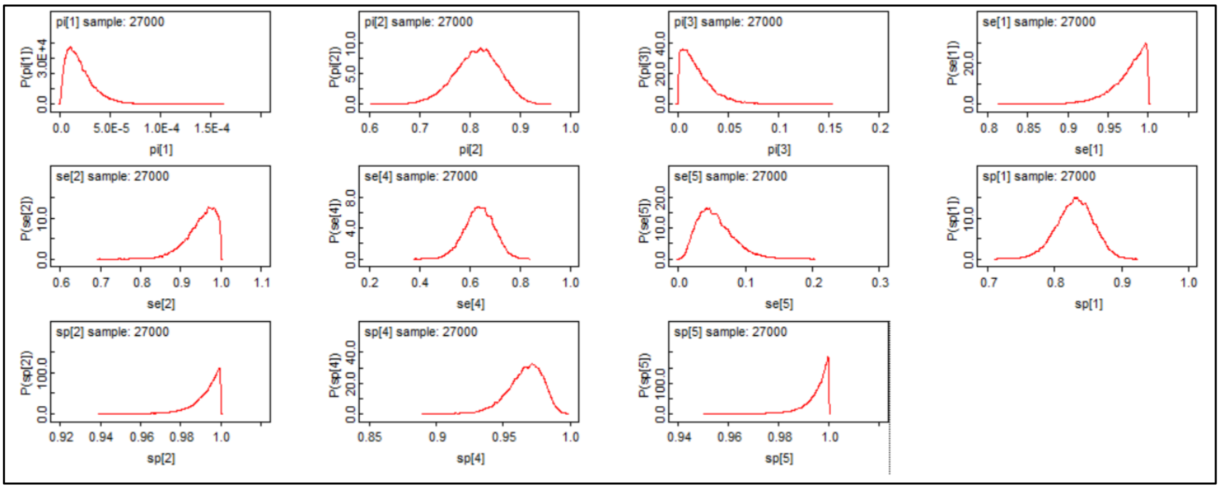

Note: pi[1] = Site 1 prevalence, pi[2] = Site 2 prevalence, pi[3] = Site 3 prevalence, se[1] = DSe of PCR, se[2] = DSe of CELL, se[4] = DSe of HIST, se[5] = DSe of IHC, sp[1] = DSp of PCR, sp[2] = DSp of CELL, sp[4] = DSp of HIST, sp[5] = DSp of IHC

2.2.4 Plots of Gelman-Rubin diagnostic for Model 2

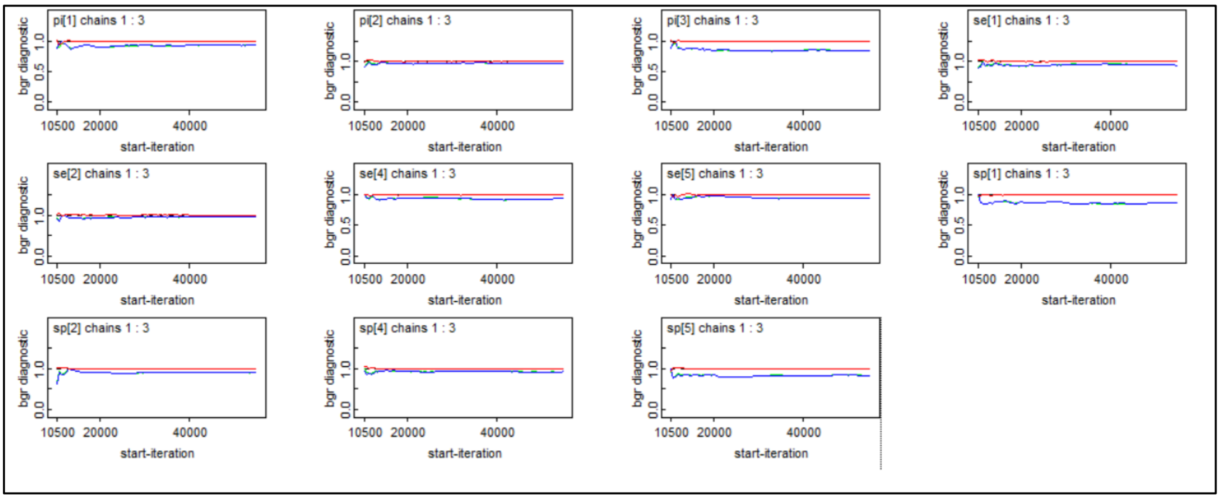

Note: pi[1] = Site 1 prevalence, pi[2] = Site 2 prevalence, pi[3] = Site 3 prevalence, se[1] = DSe of PCR, se[2] = DSe of CELL, se[4] = DSe of HIST, se[5] = DSe of IHC, sp[1] = DSp of PCR, sp[2] = DSp of CELL, sp[4] = DSp of HIST, sp[5] = DSp of IHC

2.2.5 Plots of auto-correlation for Model 2

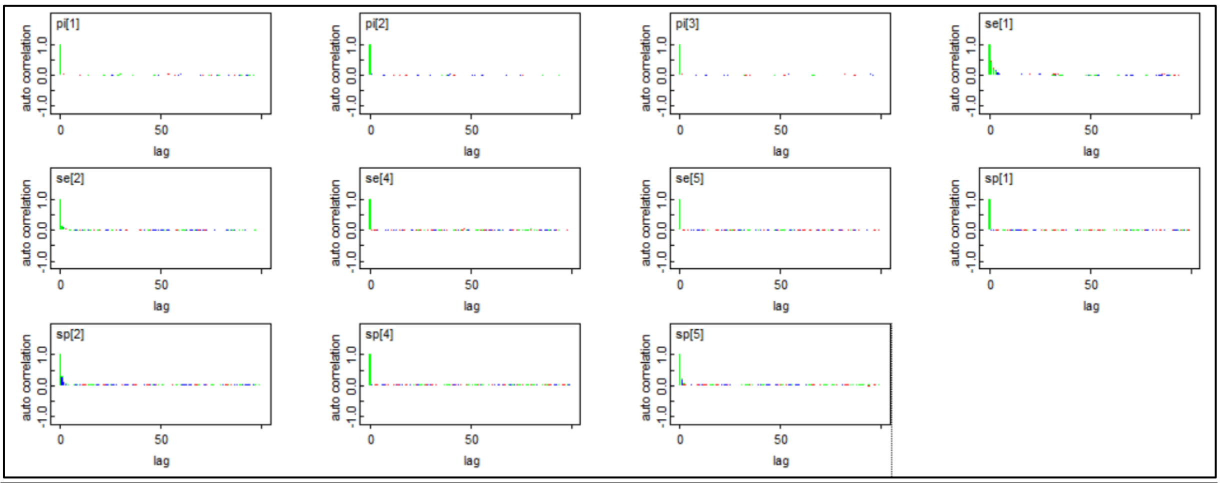

Note: pi[1] = Site 1 prevalence, pi[2] = Site 2 prevalence, pi[3] = Site 3 prevalence, se[1] = DSe of PCR, se[2] = DSe of CELL, se[4] = DSe of HIST, se[5] = DSe of IHC, sp[1] = DSp of PCR, sp[2] = DSp of CELL, sp[4] = DSp of HIST, sp[5] = DSp of IHC

3. A model with 3 populations (Site 1, Site 2, Site 3) and 4 tests (PCR, CELL, NT, HIST)

3.1. Priors of model 3

**Parameters**	**Prior distribution**
Site 1 prevalence	beta(2,100000)
Site 2 prevalence	beta(6,4)
Site 3 prevalence	beta(1,1)
DSe of all tests	beta(1,1)
DSe of all tests	beta(1,1)

3.2. MCMC Results

3.2.1.Node statistics of model 3

model	populati	test	paramete	mean	sd	MC_err	val2.5p	median	val97.5	start	sample
3	1,2,3	PCR, CELL, NT, HIST	bayesp	0.007259	0.08489	5.57E-04	0	0	0	10000	27000
3	1,2,3	PCR, CELL, NT, HIST	pi[1]	1.99E-05	1.40E-05	7.81E-08	2.52E-06	1.67E-05	5.55E-05	10000	27000
3	1,2,3	PCR, CELL, NT, HIST	pi[2]	0.8208	0.04583	2.95E-04	0.7254	0.8229	0.9044	10000	27000
3	1,2,3	PCR, CELL, NT, HIST	pi[3]	0.0146	0.01356	8.07E-05	4.01E-04	0.01076	0.05021	10000	27000
3	1,2,3	PCR, CELL, NT, HIST	se[1]	0.9718	0.02337	2.58E-04	0.9121	0.9776	0.9989	10000	27000
3	1,2,3	PCR, CELL, NT, HIST	se[2]	0.9437	0.03761	2.98E-04	0.8554	0.9499	0.996	10000	27000
3	1,2,3	PCR, CELL, NT, HIST	se[3]	0.0903	0.03467	2.05E-04	0.0345	0.08623	0.1684	10000	27000
3	1,2,3	PCR, CELL, NT, HIST	se[4]	0.6419	0.05879	3.37E-04	0.5236	0.6438	0.7522	10000	27000
3	1,2,3	PCR, CELL, NT, HIST	sp[1]	0.8306	0.02725	1.71E-04	0.7736	0.8316	0.8811	10000	27000
3	1,2,3	PCR, CELL, NT, HIST	sp[2]	0.9914	0.007067	5.45E-05	0.9733	0.9931	0.9996	10000	27000
3	1,2,3	PCR, CELL, NT, HIST	sp[3]	0.7433	0.0311	1.89E-04	0.6797	0.7445	0.8018	10000	27000
3	1,2,3	PCR, CELL, NT, HIST	sp[4]	0.9685	0.01262	7.83E-05	0.9395	0.97	0.9884	10000	27000

Note: pi[1] = Site 1 prevalence, pi[2] = Site 2 prevalence, pi[3] = Site 3 prevalence, se[1] = DSe of PCR, se[2] = DSe of CELL, se[3] = DSe of NT, se[4] = DSe of HIST, sp[1] = DSp of PCR, sp[2] = DSp of CELL, sp[3] = DSp of NT, sp[4] = DSp of HIST

3.2.2.Density plots of posterior distributions of model 3

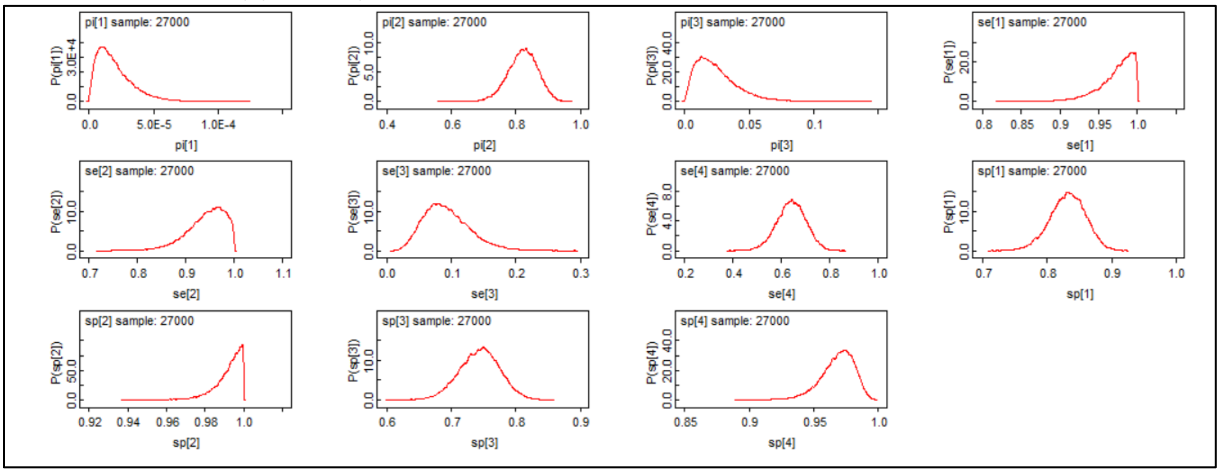

Note: pi[1] = Site 1 prevalence, pi[2] = Site 2 prevalence, pi[3] = Site 3 prevalence, se[1] = DSe of PCR, se[2] = DSe of CELL, se[3] = DSe of NT, se[4] = DSe of HIST, sp[1] = DSp of PCR, sp[2] = DSp of CELL, sp[3] = DSp of NT, sp[4] = DSp of HIST

3.2.3.Plots of Gelman-Rubin diagnostic of model 3

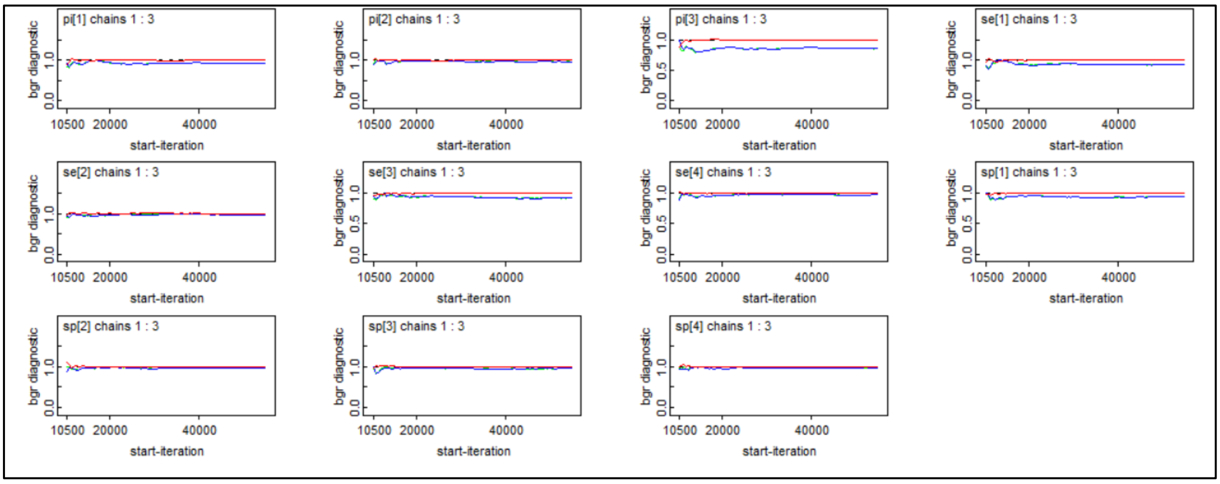

Note: pi[1] = Site 1 prevalence, pi[2] = Site 2 prevalence, pi[3] = Site 3 prevalence, se[1] = DSe of PCR, se[2] = DSe of CELL, se[3] = DSe of NT, se[4] = DSe of HIST, sp[1] = DSp of PCR, sp[2] = DSp of CELL, sp[3] = DSp of NT, sp[4] = DSp of HIST

3.2.4.Plots of auto-correlation for Model 3

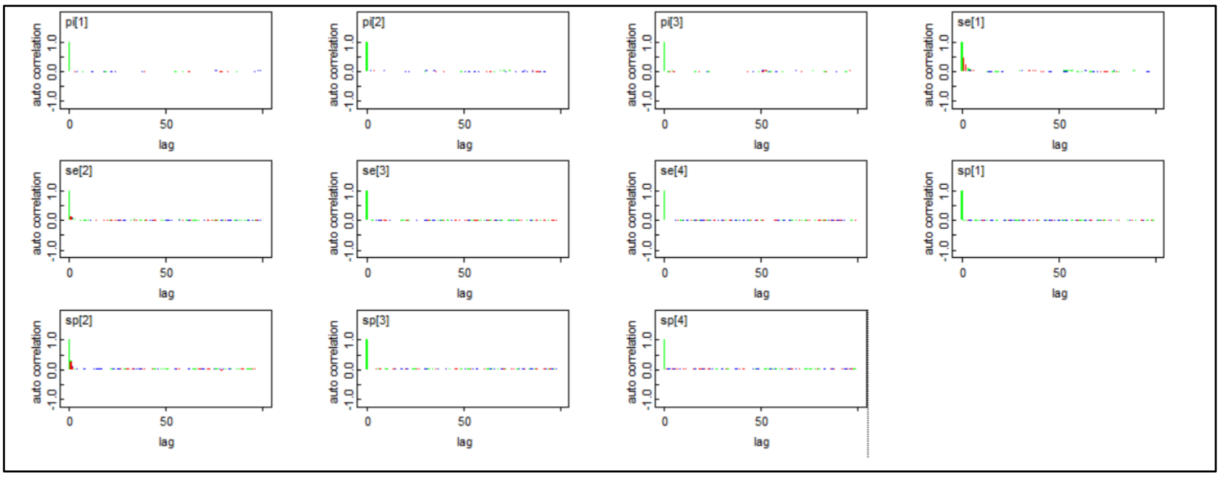

Note: pi[1] = Site 1 prevalence, pi[2] = Site 2 prevalence, pi[3] = Site 3 prevalence, se[1] = DSe of PCR, se[2] = DSe of CELL, se[3] = DSe of NT, se[4] = DSe of HIST, sp[1] = DSp of PCR, sp[2] = DSp of CELL, sp[3] = DSp of NT, sp[4] = DSp of HIST

4. A model with 3 populations (Site 1, Site 2, Site 3) and 3 tests (PCR, CELL, HIST) – This is model 4 in the paper

4.1. Priors of model 4

**Parameters**	**Prior distribution**
Site 1 prevalence	beta(2,100000)
Site 2 prevalence	beta(6,4)
Site 3 prevalence	beta(1,1)
DSe of all tests	beta(1,1)
DSe of all tests	beta(1,1)

4.2. MCMC Results

4.2.1.Node statistics of model 4

model	populati	test	paramete	mean	sd	MC_err	val2.5p	median	val97.5	start	sample
4	1,2,3	PCR, CELL,HIST	bayesp	0.03874	0.193	0.001167	0	0	1	10000	27000
4	1,2,3	PCR, CELL,HIST	pi[1]	1.99E-05	1.42E-05	8.91E-08	2.34E-06	1.67E-05	5.56E-05	10000	27000
4	1,2,3	PCR, CELL,HIST	pi[2]	0.815	0.04513	3.02E-04	0.7205	0.8171	0.8972	10000	27000
4	1,2,3	PCR, CELL,HIST	pi[3]	0.01931	0.01631	1.04E-04	7.44E-04	0.01518	0.0616	10000	27000
4	1,2,3	PCR, CELL,HIST	se[1]	0.9737	0.02245	2.20E-04	0.917	0.9796	0.999	10000	27000
4	1,2,3	PCR, CELL,HIST	se[2]	0.9481	0.03738	2.83E-04	0.8581	0.9554	0.9971	10000	27000
4	1,2,3	PCR, CELL,HIST	se[3]	0.6437	0.05823	3.67E-04	0.5274	0.6447	0.7543	10000	27000
4	1,2,3	PCR, CELL,HIST	sp[1]	0.8305	0.0275	1.71E-04	0.7737	0.8315	0.8814	10000	27000
4	1,2,3	PCR, CELL,HIST	sp[2]	0.9928	0.006585	5.51E-05	0.9751	0.9946	0.9998	10000	27000
4	1,2,3	PCR, CELL,HIST	sp[3]	0.9687	0.01271	7.97E-05	0.9398	0.9702	0.9889	10000	27000

Note: pi[1] = Site 1 prevalence, pi[2] = Site 2 prevalence, pi[3] = Site 3 prevalence, se[1] = DSe of PCR, se[2] = DSe of CELL, se[3] = DSe of HIST, sp[1] = DSp of PCR, sp[2] = DSp of CELL, sp[3] = DSp of HIST

4.2.2. Density plots of posteriors model 4

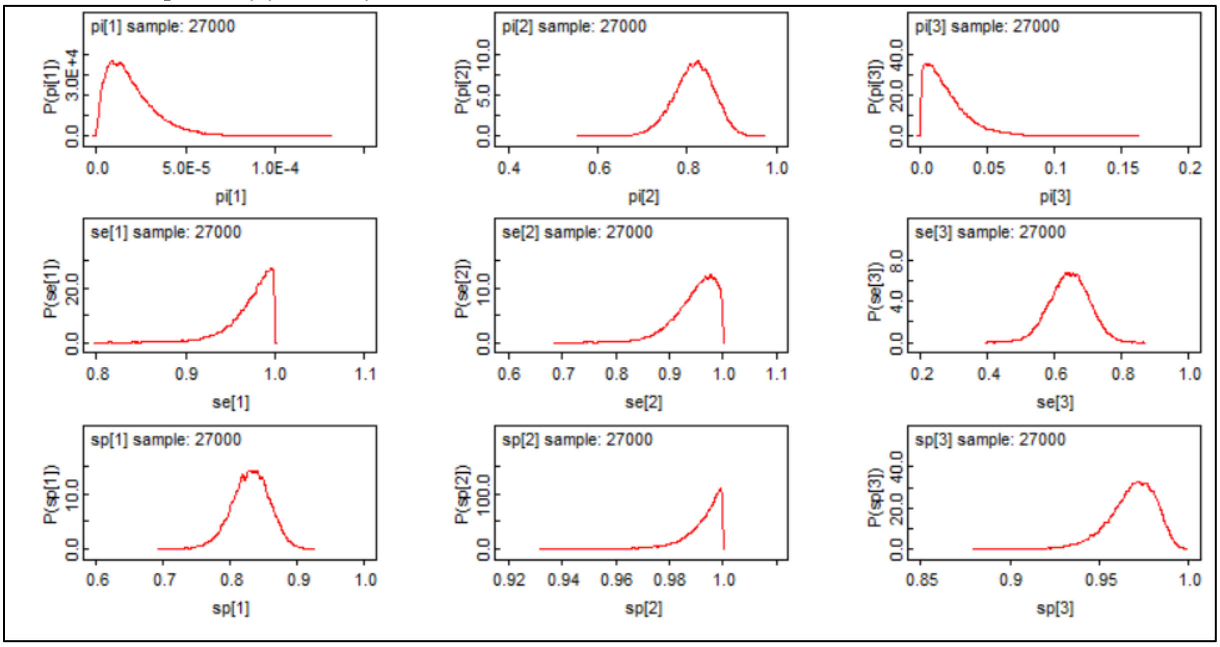

Note: pi[1] = Site 1 prevalence, pi[2] = Site 2 prevalence, pi[3] = Site 3 prevalence, se[1] = DSe of PCR, se[2] = DSe of CELL, se[3] = DSe of HIST, sp[1] = DSp of PCR, sp[2] = DSp of CELL, sp[3] = DSp of HIST

4.2.3.Plots of Gelman-Rubin diagnostic of model 4

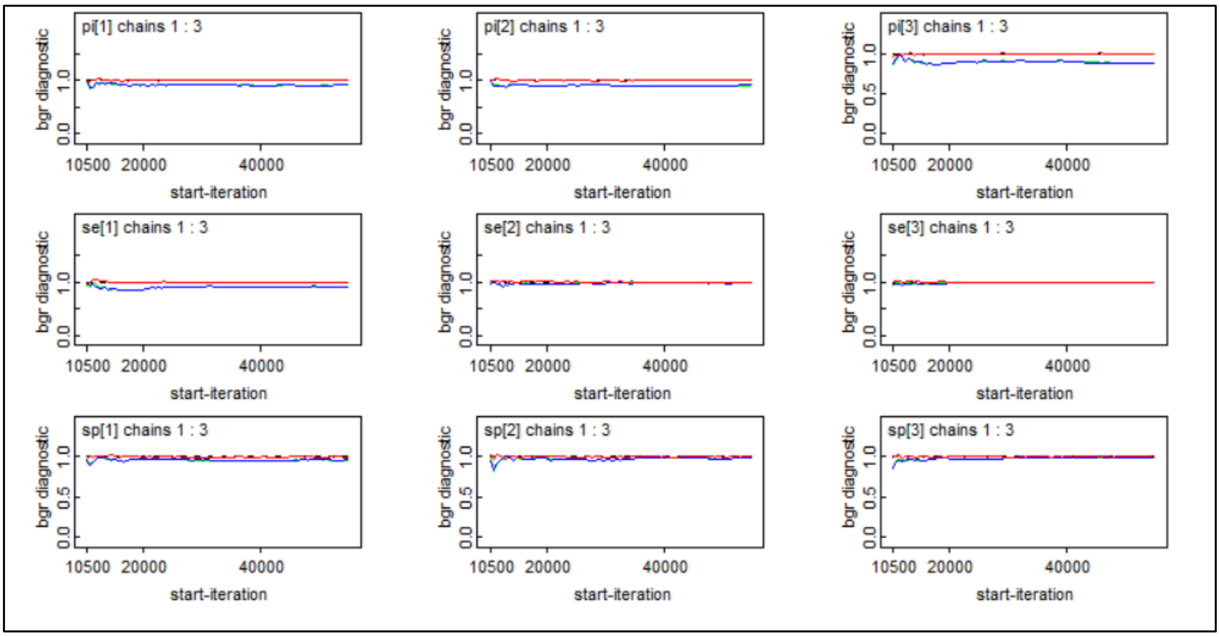

Note: pi[1] = Site 1 prevalence, pi[2] = Site 2 prevalence, pi[3] = Site 3 prevalence, se[1] = DSe of PCR, se[2] = DSe of CELL, se[3] = DSe of HIST, sp[1] = DSp of PCR, sp[2] = DSp of CELL, sp[3] = DSp of HIST

4.2.4.Plots of auto-correlation of model 4

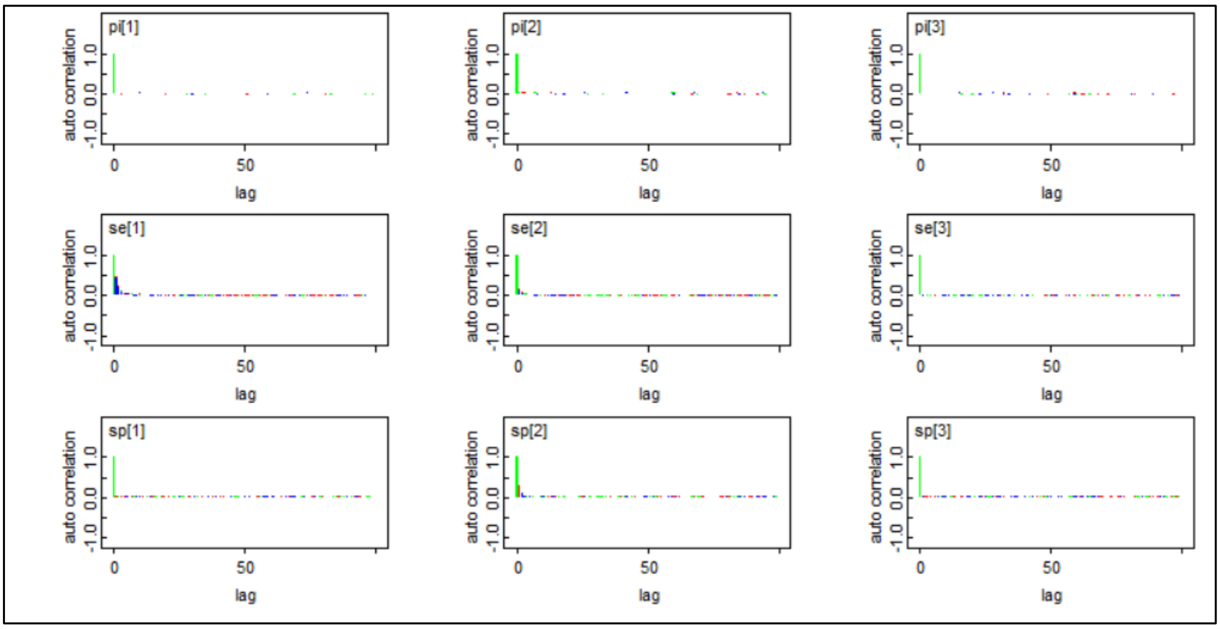

Note: pi[1] = Site 1 prevalence, pi[2] = Site 2 prevalence, pi[3] = Site 3 prevalence, se[1] = DSe of PCR, se[2] = DSe of CELL, se[3] = DSe of HIST, sp[1] = DSp of PCR, sp[2] = DSp of CELL, sp[3] = DSp of HIST

5. A model with 2 populations (Site 1, Site 2) and 5 tests (PCR, CELL, NT, HIST, IHC)

5.1. Priors of model parameters of model 5

**Parameters**	**Prior distribution**
Site 1 prevalence	beta(2, 100000)
Site 2 prevalence	beta(6,4)
DSe of all tests	beta(1,1)
DSp of all tests	beta(1,1)

5.2. MCMC Results

5.2.1.Node statistics of model of 5

model	populati	test	paramete	mean	sd	MC_err	val2.5p	median	val97.5	start	sample
5	1 and 2	PCR, CELL, NT, HIST, IHC	bayesp	0.4818	0.4997	0.002946	0	0	1	10000	27000
5	1 and 2	PCR, CELL, NT, HIST, IHC	pi[1]	2.01E-05	1.42E-05	7.66E-08	2.42E-06	1.69E-05	5.64E-05	10000	27000
5	1 and 2	PCR, CELL, NT, HIST, IHC	pi[2]	0.8645	0.03872	2.44E-04	0.781	0.8674	0.9314	10000	27000
5	1 and 2	PCR, CELL, NT, HIST, IHC	se[1]	0.9713	0.02206	2.04E-04	0.9159	0.9763	0.9984	10000	27000
5	1 and 2	PCR, CELL, NT, HIST, IHC	se[2]	0.8935	0.03804	2.56E-04	0.8102	0.8967	0.9584	10000	27000
5	1 and 2	PCR, CELL, NT, HIST, IHC	se[3]	0.08056	0.0315	1.86E-04	0.03026	0.07688	0.1529	10000	27000
5	1 and 2	PCR, CELL, NT, HIST, IHC	se[4]	0.6075	0.05607	3.23E-04	0.4975	0.608	0.7168	10000	27000
5	1 and 2	PCR, CELL, NT, HIST, IHC	se[5]	0.05275	0.02545	1.52E-04	0.01477	0.04889	0.113	10000	27000
5	1 and 2	PCR, CELL, NT, HIST, IHC	sp[1]	0.9829	0.01614	1.22E-04	0.9395	0.9876	0.9995	10000	27000
5	1 and 2	PCR, CELL, NT, HIST, IHC	sp[2]	0.988	0.01134	8.54E-05	0.9577	0.9913	0.9997	10000	27000
5	1 and 2	PCR, CELL, NT, HIST, IHC	sp[3]	0.99	0.009912	6.03E-05	0.9635	0.9931	0.9997	10000	27000
5	1 and 2	PCR, CELL, NT, HIST, IHC	sp[4]	0.9856	0.01283	9.65E-05	0.9526	0.9891	0.9996	10000	27000
5	1 and 2	PCR, CELL, NT, HIST, IHC	sp[5]	0.9883	0.01095	7.64E-05	0.9591	0.9915	0.9997	10000	27000

Note: pi[1] = Site 1 prevalence, pi[2] = Site 2 prevalence, se[1] = DSe of PCR, se[2] = DSe of CELL, se[3] = DSe of NT, se[4] = DSe of HIST, se[5] = DSe of IHC, sp[1] = DSp of PCR, sp[2] = DSp of CELL, sp[3] = DSp of NT, sp[4] = DSp of HIST, sp[5] = DSp of IHC

5.2.2.Density plot of posteriors of model of 5

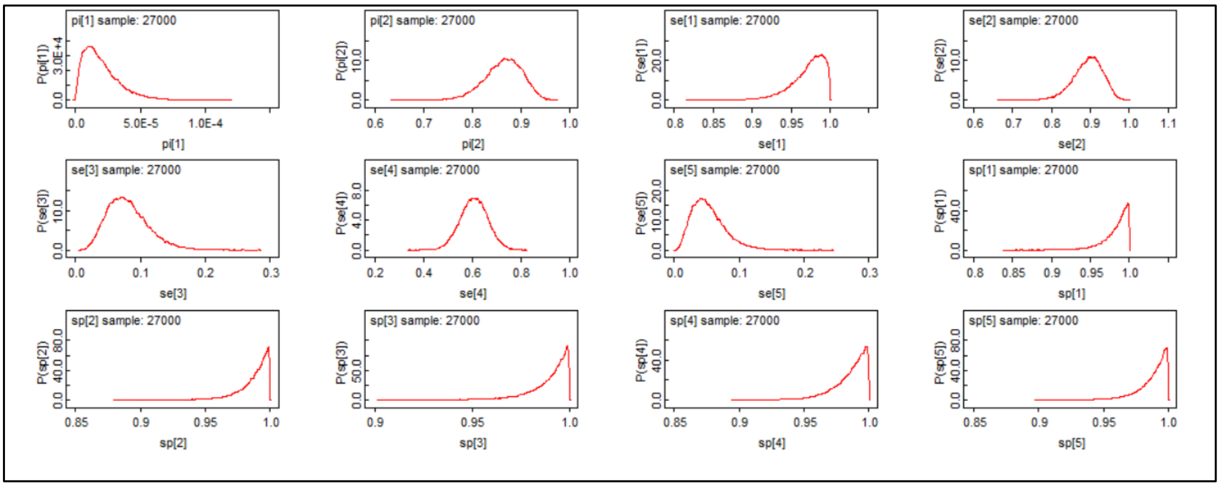

Note: pi[1] = Site 1 prevalence, pi[2] = Site 2 prevalence, se[1] = DSe of PCR, se[2] = DSe of CELL, se[3] = DSe of NT, se[4] = DSe of HIST, se[5] = DSe of IHC, sp[1] = DSp of PCR, sp[2] = DSp of CELL, sp[3] = DSp of NT, sp[4] = DSp of HIST, sp[5] = DSp of IHC

5.2.3.Plots of Gelman-Rubin diagnostic of model 5

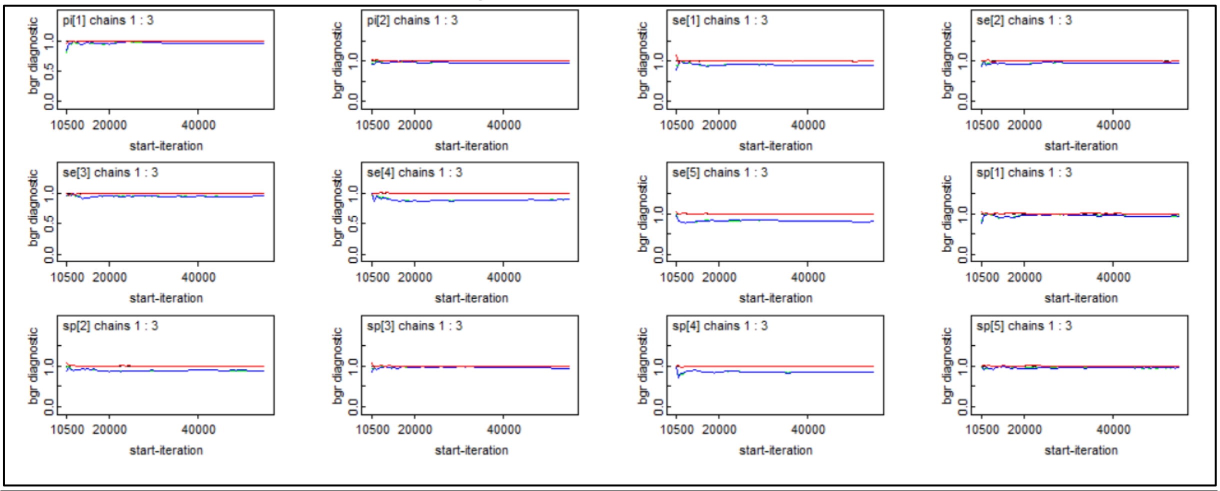

Note: pi[1] = Site 1 prevalence, pi[2] = Site 2 prevalence, se[1] = DSe of PCR, se[2] = DSe of CELL, se[3] = DSe of NT, se[4] = DSe of HIST, se[5] = DSe of IHC, sp[1] = DSp of PCR, sp[2] = DSp of CELL, sp[3] = DSp of NT, sp[4] = DSp of HIST, sp[5] = DSp of IHC

5.2.4.Plots of auto correlation of model 5

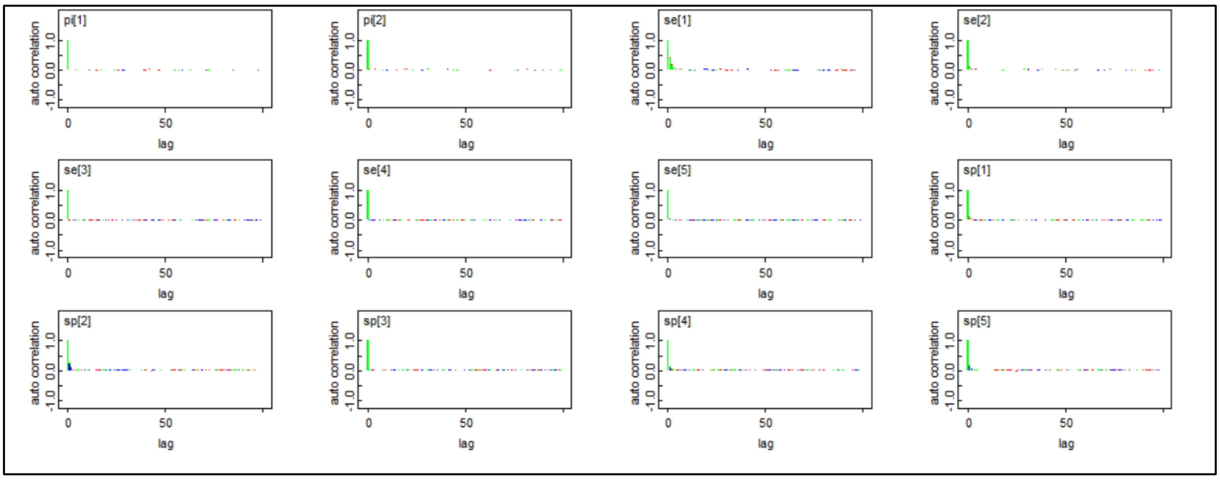

Note: pi[1] = Site 1 prevalence, pi[2] = Site 2 prevalence, se[1] = DSe of PCR, se[2] = DSe of CELL, se[3] = DSe of NT, se[4] = DSe of HIST, se[5] = DSe of IHC, sp[1] = DSp of PCR, sp[2] = DSp of CELL, sp[3] = DSp of NT, sp[4] = DSp of HIST, sp[5] = DSp of IHC

6. A model with 2 populations (Site 2, Site 3) and 5 tests (PCR, CELL, NT, HIST, IHC)

6.1. Priors of model parameters of model 6

Parameters	Prior distribution
Site 2 prevalence	beta(6,4)
Site 3 prevalence	beta(1,1)
DSe of all tests	beta(1,1)
DSp of all tests	beta(1,1)

6.2. MCMC Results

6.2.1.Node statistics of model 6

model	populati	test	paramete	mean	sd	MC_err	val2.5p	median	val97.5	start	sample
6	2 and 3	PCR, CELL, NT, HIST, IHC	bayesp	0.2376	0.4256	0.002566	0	0	1	10000	27000
6	2 and 3	PCR, CELL, NT, HIST, IHC	pi[1]	0.818	0.04673	3.38E-04	0.7225	0.8196	0.9052	10000	27000
6	2 and 3	PCR, CELL, NT, HIST, IHC	pi[2]	0.01186	0.01167	7.15E-05	3.15E-04	0.008289	0.04301	10000	27000
6	2 and 3	PCR, CELL, NT, HIST, IHC	se[1]	0.9668	0.02721	3.56E-04	0.8983	0.9734	0.9987	10000	27000
6	2 and 3	PCR, CELL, NT, HIST, IHC	se[2]	0.9476	0.03748	3.90E-04	0.8551	0.955	0.997	10000	27000
6	2 and 3	PCR, CELL, NT, HIST, IHC	se[3]	0.08637	0.03382	2.06E-04	0.03222	0.08226	0.1637	10000	27000
6	2 and 3	PCR, CELL, NT, HIST, IHC	se[4]	0.6402	0.05866	3.89E-04	0.5227	0.6415	0.7505	10000	27000
6	2 and 3	PCR, CELL, NT, HIST, IHC	se[5]	0.05576	0.02696	1.65E-04	0.01564	0.05178	0.119	10000	27000
6	2 and 3	PCR, CELL, NT, HIST, IHC	sp[1]	0.6856	0.04491	2.66E-04	0.5944	0.6866	0.7706	10000	27000
6	2 and 3	PCR, CELL, NT, HIST, IHC	sp[2]	0.9819	0.01353	1.02E-04	0.9476	0.9849	0.9987	10000	27000
6	2 and 3	PCR, CELL, NT, HIST, IHC	sp[3]	0.5511	0.03424	2.18E-04	0.5025	0.546	0.6284	10000	27000
6	2 and 3	PCR, CELL, NT, HIST, IHC	sp[4]	0.9378	0.0228	1.40E-04	0.8869	0.9403	0.9748	10000	27000
6	2 and 3	PCR, CELL, NT, HIST, IHC	sp[5]	0.9911	0.008887	6.11E-05	0.9671	0.9938	0.9998	10000	27000

Note: pi[1] = Site 2 prevalence, pi[2] = Site 3 prevalence, se[1] = DSe of PCR, se[2] = DSe of CELL, se[3] = DSe of NT, se[4] = DSe of HIST, se[5] = DSe of IHC, sp[1] = DSp of PCR, sp[2] = DSp of CELL, sp[3] = DSp of NT, sp[4] = DSp of HIST, sp[5] = DSp of IHC

6.2.2.Density plots of posterior distributions of model 6

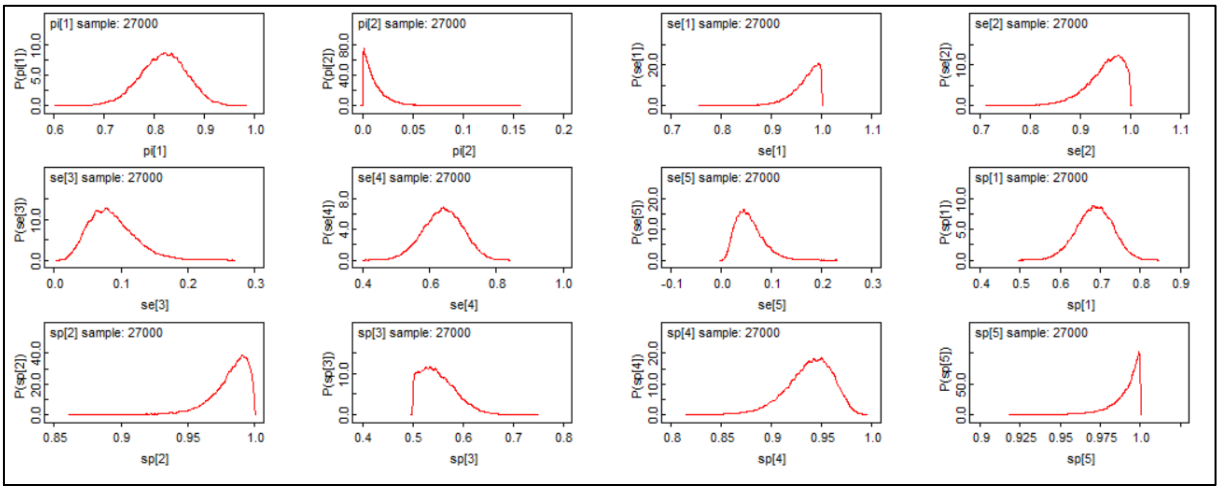

Note: pi[1] = Site 2 prevalence, pi[2] = Site 3 prevalence, se[1] = DSe of PCR, se[2] = DSe of CELL, se[3] = DSe of NT, se[4] = DSe of HIST, se[5] = DSe of IHC, sp[1] = DSp of PCR, sp[2] = DSp of CELL, sp[3] = DSp of NT, sp[4] = DSp of HIST, sp[5] = DSp of IHC

6.2.3. Plots of Gelman-Rubin statistics for Model 6

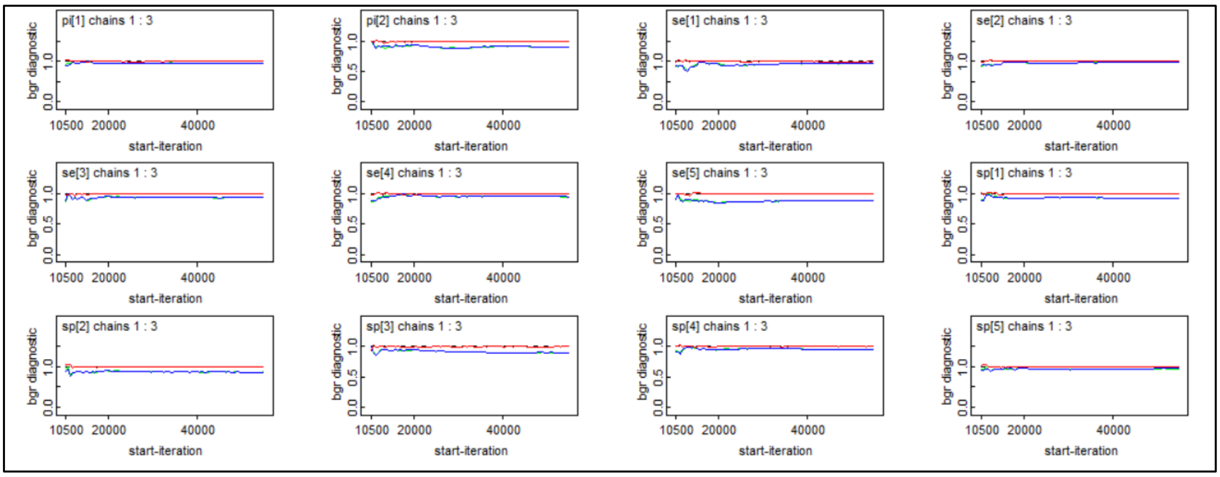

Note: pi[1] = Site 2 prevalence, pi[2] = Site 3 prevalence, se[1] = DSe of PCR, se[2] = DSe of CELL, se[3] = DSe of NT, se[4] = DSe of HIST, se[5] = DSe of IHC, sp[1] = DSp of PCR, sp[2] = DSp of CELL, sp[3] = DSp of NT, sp[4] = DSp of HIST, sp[5] = DSp of IHC

6.2.4. Plots of auto-correlation for Model 6

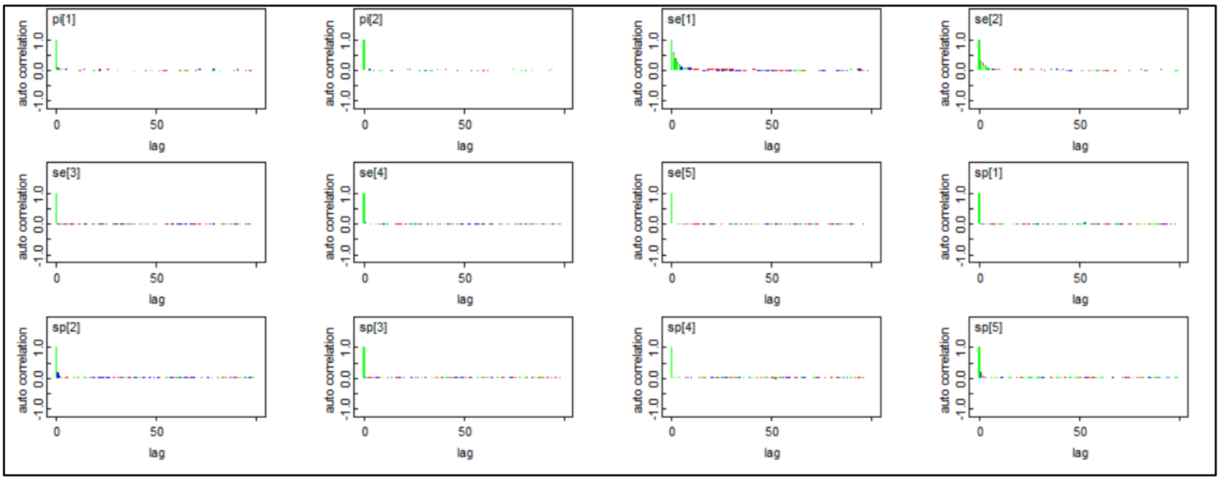

Note: pi[1] = Site 2 prevalence, pi[2] = Site 3 prevalence, se[1] = DSe of PCR, se[2] = DSe of CELL, se[3] = DSe of NT, se[4] = DSe of HIST, se[5] = DSe of IHC, sp[1] = DSp of PCR, sp[2] = DSp of CELL, sp[3] = DSp of NT, sp[4] = DSp of HIST, sp[5] = DSp of IHC

7. A model with 2 populations (Site 1, Site 3) and 5 tests (PCR, CELL, NT, HIST, IHC)

7.1. Priors of model parameters of model 7

Parameters	Prior distribution
Site 1 prevalence	beta(2,100000)
Site 3 prevalence	beta(1,1)
DSe of all tests	beta(1,1)
DSp of all tests	beta(1,1)

7.2. MCMC Results

7.2.1. Node statistics of model 7

model	populati	test	paramete	mean	sd	MC_err	val2.5p	median	val97.5	start	sample
7	1 and 3	PCR, CELL, NT, HIST, IHC	bayesp	0.8232	0.3815	0.002257	0	1	1	10000	27000
7	1 and 3	PCR, CELL, NT, HIST, IHC	pi[1]	2.00E-05	1.42E-05	8.00E-08	2.43E-06	1.68E-05	5.57E-05	10000	27000
7	1 and 3	PCR, CELL, NT, HIST, IHC	pi[2]	0.5605	0.05577	3.45E-04	0.4519	0.5602	0.6711	10000	27000
7	1 and 3	PCR, CELL, NT, HIST, IHC	se[1]	0.526	0.06796	4.27E-04	0.3933	0.5258	0.6592	10000	27000
7	1 and 3	PCR, CELL, NT, HIST, IHC	se[2]	0.03512	0.0243	1.70E-04	0.004321	0.02975	0.09572	10000	27000
7	1 and 3	PCR, CELL, NT, HIST, IHC	se[3]	0.9216	0.04977	3.06E-04	0.8034	0.9297	0.993	10000	27000
7	1 and 3	PCR, CELL, NT, HIST, IHC	se[4]	0.1136	0.04144	2.45E-04	0.04586	0.1093	0.2058	10000	27000
7	1 and 3	PCR, CELL, NT, HIST, IHC	se[5]	0.01743	0.01726	1.26E-04	4.14E-04	0.01224	0.06349	10000	27000
7	1 and 3	PCR, CELL, NT, HIST, IHC	sp[1]	0.9876	0.01094	7.49E-05	0.9593	0.9905	0.9996	10000	27000
7	1 and 3	PCR, CELL, NT, HIST, IHC	sp[2]	0.9913	0.008342	5.91E-05	0.9691	0.9937	0.9998	10000	27000
7	1 and 3	PCR, CELL, NT, HIST, IHC	sp[3]	0.9889	0.01107	6.39E-05	0.9592	0.9923	0.9997	10000	27000
7	1 and 3	PCR, CELL, NT, HIST, IHC	sp[4]	0.991	0.008388	6.78E-05	0.969	0.9934	0.9997	10000	27000
7	1 and 3	PCR, CELL, NT, HIST, IHC	sp[5]	0.991	0.008322	6.23E-05	0.9693	0.9934	0.9997	10000	27000

Note: pi[1] = Site 1 prevalence, pi[2] = Site 3 prevalence, se[1] = DSe of PCR, se[2] = DSe of CELL, se[3] = DSe of NT, se[4] = DSe of HIST, se[5] = DSe of IHC, sp[1] = DSp of PCR, sp[2] = DSp of CELL, sp[3] = DSp of NT, sp[4] = DSp of HIST, sp[5] = DSp of IHC

7.2.2. Density plots of posterior distributions of model 7

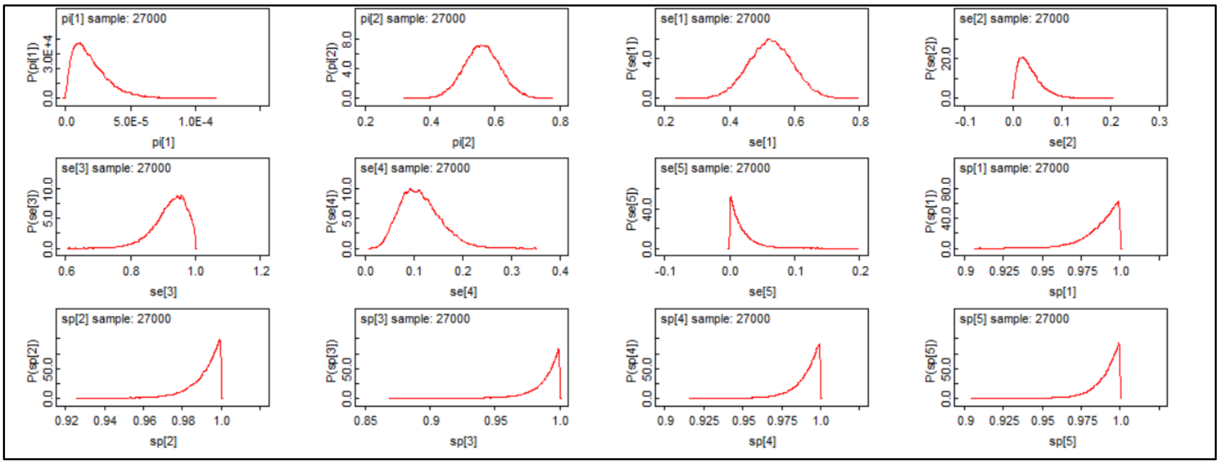

Note: pi[1] = Site 1 prevalence, pi[2] = Site 3 prevalence, se[1] = DSe of PCR, se[2] = DSe of CELL, se[3] = DSe of NT, se[4] = DSe of HIST, se[5] = DSe of IHC, sp[1] = DSp of PCR, sp[2] = DSp of CELL, sp[3] = DSp of NT, sp[4] = DSp of HIST, sp[5] = DSp of IHC

7.2.3.Plots of Gelman-Rubin statistics for Model 7

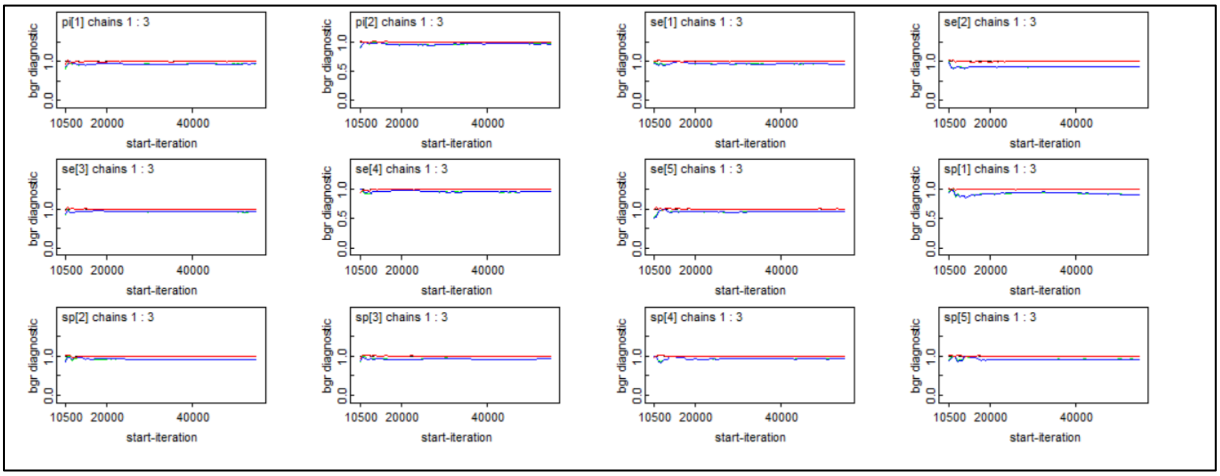

Note: pi[1] = Site 1 prevalence, pi[2] = Site 3 prevalence, se[1] = DSe of PCR, se[2] = DSe of CELL, se[3] = DSe of NT, se[4] = DSe of HIST, se[5] = DSe of IHC, sp[1] = DSp of PCR, sp[2] = DSp of CELL, sp[3] = DSp of NT, sp[4] = DSp of HIST, sp[5] = DSp of IHC

7.2.4.Plots of auto-correlation for Model 7

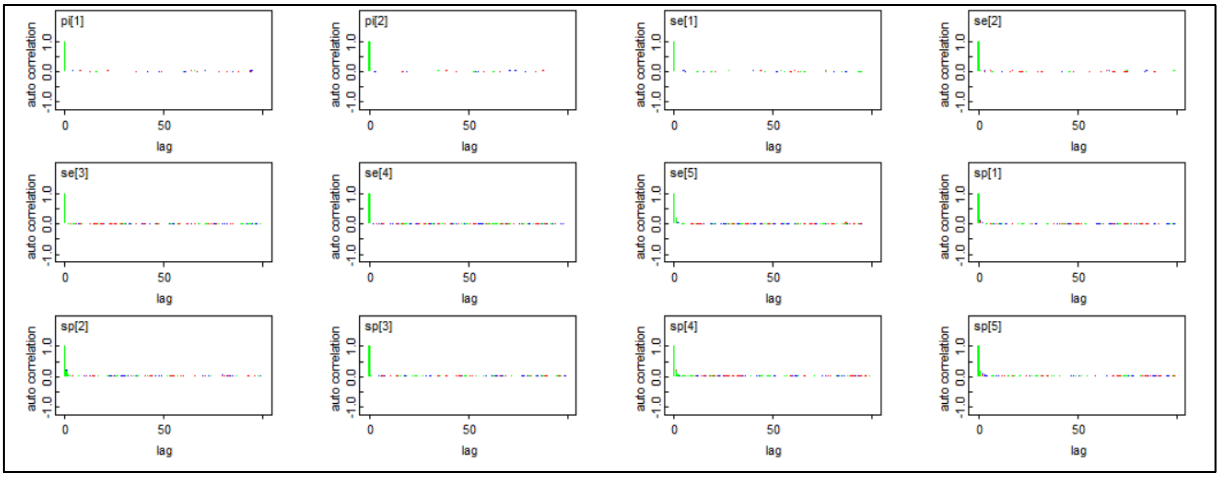

Note: pi[1] = Site 1 prevalence, pi[2] = Site 3 prevalence, se[1] = DSe of PCR, se[2] = DSe of CELL, se[3] = DSe of NT, se[4] = DSe of HIST, se[5] = DSe of IHC, sp[1] = DSp of PCR, sp[2] = DSp of CELL, sp[3] = DSp of NT, sp[4] = DSp of HIST, sp[5] = DSp of IHC

Step function results

Table 1. Probability that DSe of Test 1 is greater than DSe of test 2. Bold indicated pairs that not a significant difference (mean of probability > 0.95 or < 0.05)

Test1	Test2	Probability Test 1 > Test 2	mean	sd	MC_error	val2.5pc	median	val97.5pc	start	sample
**PCR**	**CELL**	**Prsediff[1]**	**0.7913**	**0.4064**	**0.003268**	**0**	**1**	**1**	**5000**	**13500**
PCR	NT	Prsediff[2]	1	0	8.61E-13	1	1	1	5000	13500
PCR	HIST	Prsediff[3]	1	0	8.61E-13	1	1	1	5000	13500
PCR	IHC	Prsediff[4]	1	0	8.61E-13	1	1	1	5000	13500
**CELL**	**PCR**	**Prsediff[5]**	**0.2087**	**0.4064**	**0.003268**	**0**	**0**	**1**	**5000**	**13500**
CELL	NT	Prsediff[6]	1	0	8.61E-13	1	1	1	5000	13500
CELL	HIST	Prsediff[7]	1	0	8.61E-13	1	1	1	5000	13500
CELL	IHC	Prsediff[8]	1	0	8.61E-13	1	1	1	5000	13500
NT	PCR	Prsediff[9]	0	0	8.61E-13	0	0	0	5000	13500
NT	CELL	Prsediff[10]	0	0	8.61E-13	0	0	0	5000	13500
NT	HIST	Prsediff[11]	0	0	8.61E-13	0	0	0	5000	13500
**NT**	**IHC**	**Prsediff[12]**	**0.7916**	**0.4062**	**0.003674**	**0**	**1**	**1**	**5000**	**13500**
HIST	PCR	Prsediff[13]	0	0	8.61E-13	0	0	0	5000	13500
HIST	CELL	Prsediff[14]	0	0	8.61E-13	0	0	0	5000	13500
HIST	NT	Prsediff[15]	1	0	8.61E-13	1	1	1	5000	13500
HIST	IHC	Prsediff[16]	1	0	8.61E-13	1	1	1	5000	13500
IHC	PCR	Prsediff[17]	0	0	8.61E-13	0	0	0	5000	13500
IHC	CELL	Prsediff[18]	0	0	8.61E-13	0	0	0	5000	13500
**IHC**	**NT**	**Prsediff[19]**	**0.2084**	**0.4062**	**0.003674**	**0**	**0**	**1**	**5000**	**13500**
IHC	HIST	Prsediff[20]	0	0	8.61E-13	0	0	0	5000	13500

Table 2. Probability that DSp of Test 1 is greater than DSp of test 2. Bold indicated pairs with a significant difference (mean > 0.95 or < 0.05)

Test1	Test2	Probability Test 1 > Test 2	mean	sd	MC_error	val2.5pc	median	val97.5pc	start	sample
PCR	CELL	Prspdiff[1]	0	0	8.61E-13	0	0	0	5000	13500
PCR	NT	Prspdiff[2]	0.9829	0.1297	0.001129	1	1	1	5000	13500
PCR	HIST	Prspdiff[3]	0	0	8.61E-13	0	0	0	5000	13500
PCR	IHC	Prspdiff[4]	0	0	8.61E-13	0	0	0	5000	13500
CELL	PCR	Prspdiff[5]	1	0	8.61E-13	1	1	1	5000	13500
CELL	NT	Prspdiff[6]	1	0	8.61E-13	1	1	1	5000	13500
CELL	HIST	Prspdiff[7]	0.969	0.1734	0.001516	0	1	1	5000	13500
**CELL**	**IHC**	**Prspdiff[8]**	**0.3158**	**0.4648**	**0.004421**	**0**	**0**	**1**	**5000**	**13500**
NT	PCR	Prspdiff[9]	0.01711	0.1297	0.001129	0	0	0	5000	13500
NT	CELL	Prspdiff[10]	0	0	8.61E-13	0	0	0	5000	13500
NT	HIST	Prspdiff[11]	0	0	8.61E-13	0	0	0	5000	13500
NT	IHC	Prspdiff[12]	0	0	8.61E-13	0	0	0	5000	13500
HIST	PCR	Prspdiff[13]	1	0	8.61E-13	1	1	1	5000	13500
HIST	CELL	Prspdiff[14]	0.03104	0.1734	0.001516	0	0	1	5000	13500
HIST	NT	Prspdiff[15]	1	0	8.61E-13	1	1	1	5000	13500
HIST	IHC	Prspdiff[16]	0.004	0.06312	5.63E-04	0	0	0	5000	13500
IHC	PCR	Prspdiff[17]	1	0	8.61E-13	1	1	1	5000	13500
**IHC**	**CELL**	**Prspdiff[18]**	**0.6842**	**0.4648**	**0.004421**	**0**	**1**	**1**	**5000**	**13500**
IHC	NT	Prspdiff[19]	1	0	8.61E-13	1	1	1	5000	13500
IHC	HIST	Prspdiff[20]	0.996	0.06312	5.63E-04	1	1	1	5000	13500

Sensitivity analysis

Figure 1. Caterpillar plots for posterior estimats of site prevalence, DSe, and DSp based on model 1 default (row 1), SA1 (row 2), and SA2 (row 3)

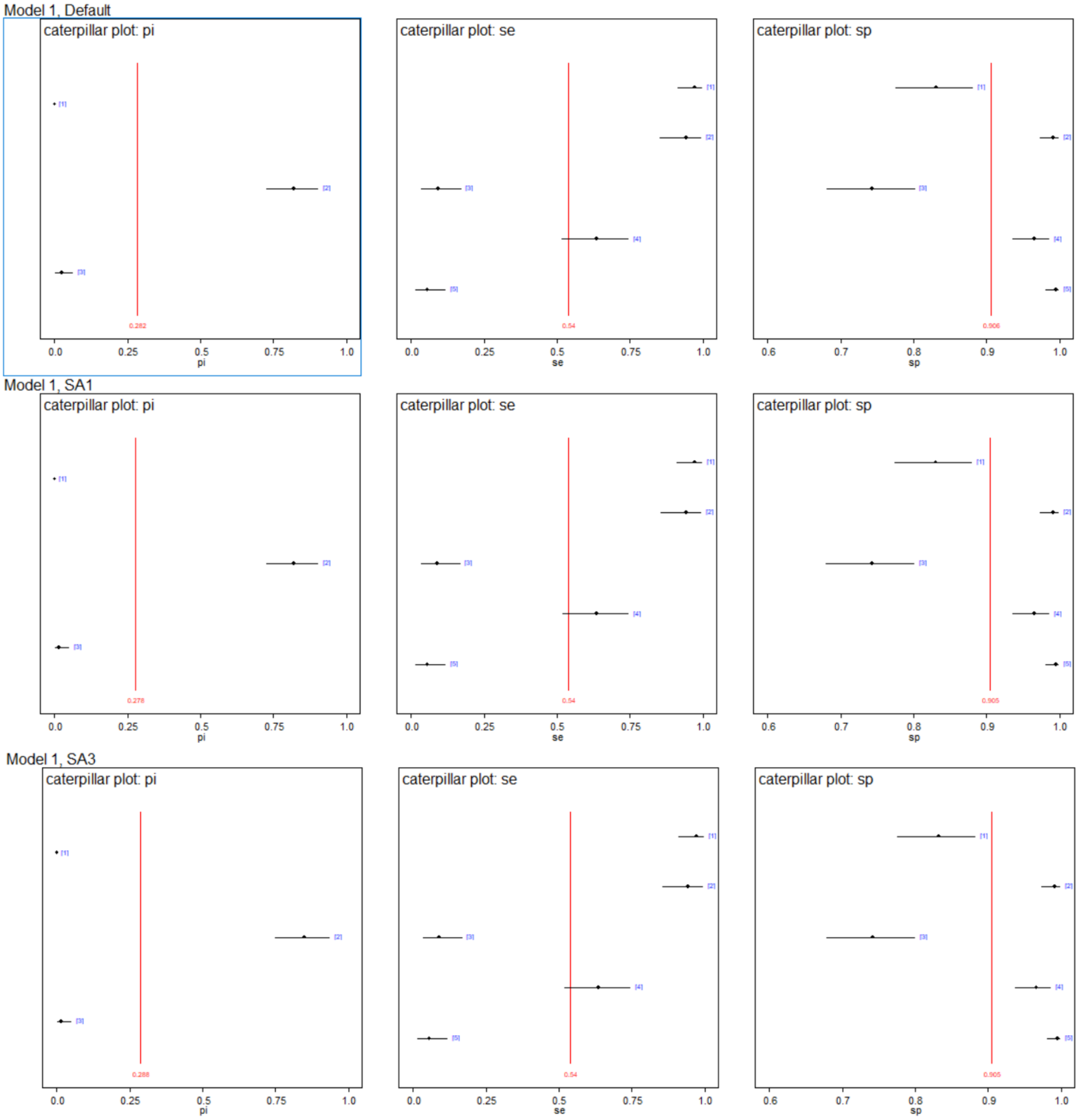

Note: pi[1] = Site 1 prevalence, pi[2] = Site 2 prevalence, pi[3] = Site 3 prevalence, se[1] = DSe of PCR, se[2] = DSe of CELL, se[3] = DSe of NT, se[4] = DSe of HIST, se[5] = DSe of IHC, sp[1] = DSp of PCR, sp[2] = DSp of CELL, sp[3] = DSp of NT, sp[4] = DSp of HIST, sp[5] = DSp of IHC

Figure 2. Caterpillar plots for posterior estimates of site prevalence, DSe, and DSp based on model 2 default, SA3, and SA4

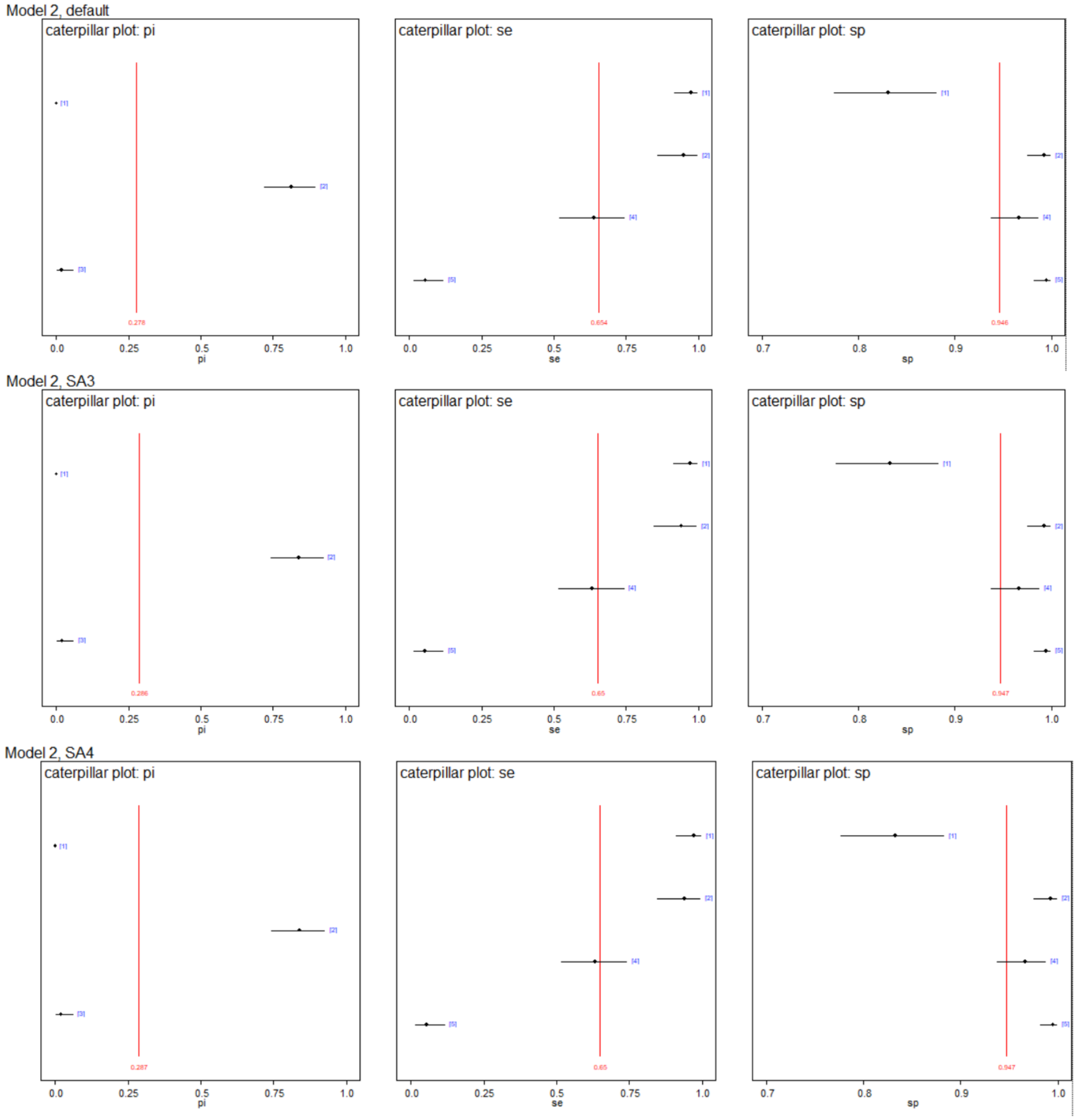

Figure 3. Caterpillar plots for posterior estimates of site prevalence, DSe, and DSp based on model 3 default, SA5, and SA6

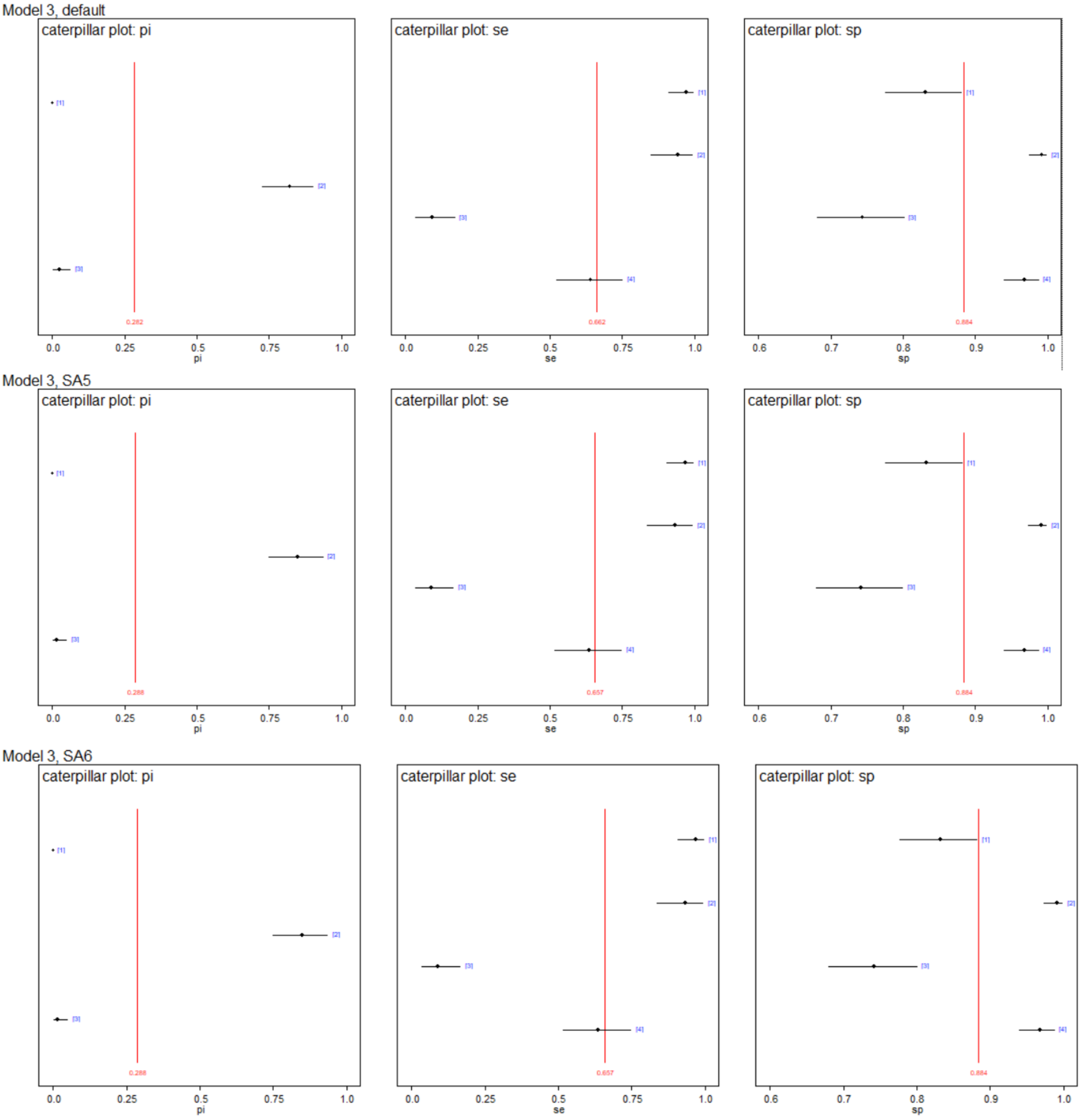

Figure 4. Caterpillar plots for posterior estimates of site prevalence, DSe, and DSp based on model 4 default, SA7, and SA8

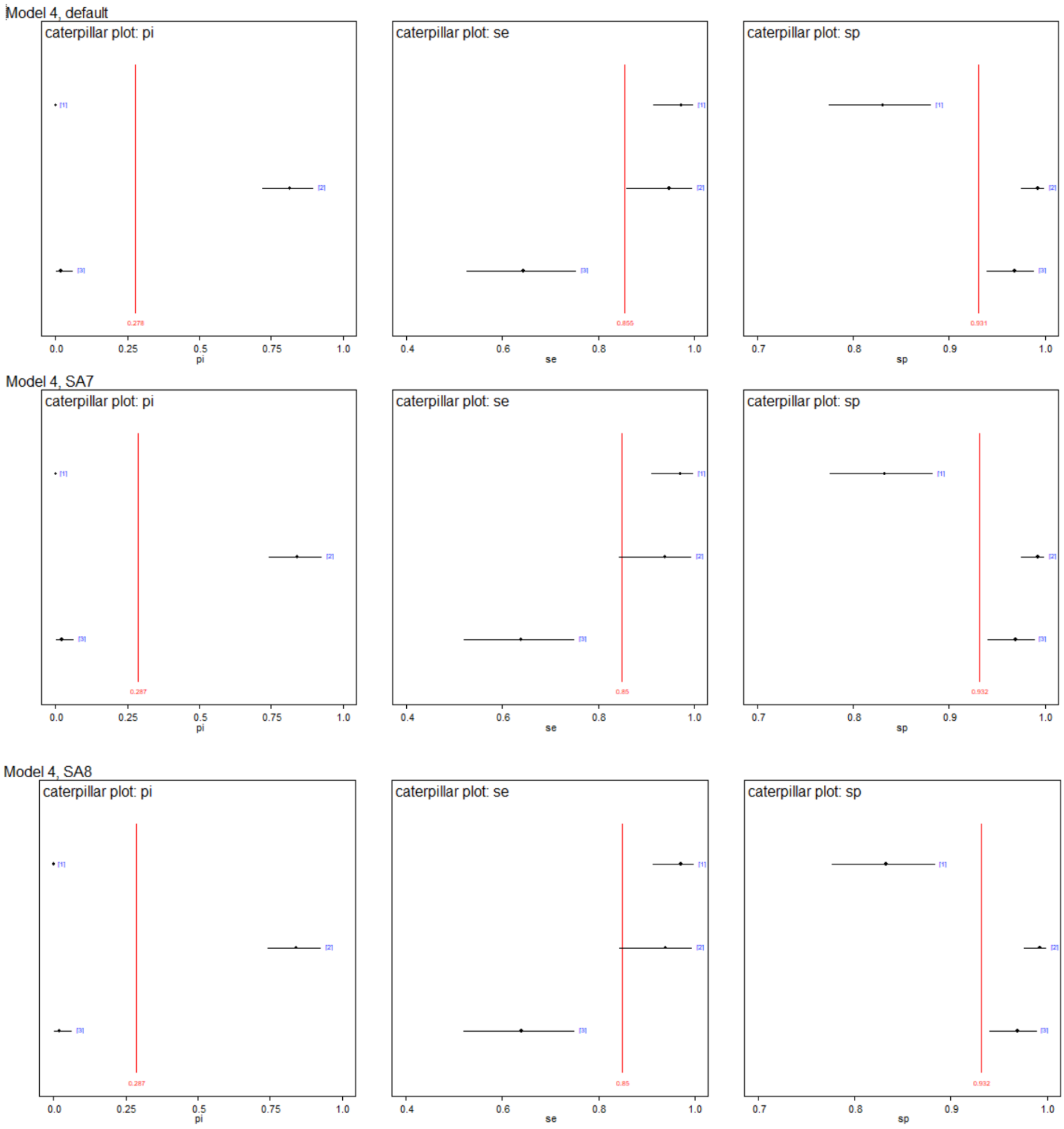

Supplement: Supplementary file 1 [file Data_Sheet_1.PDF]
